# Obesity: pathophysiology and therapeutic interventions

**DOI:** 10.1186/s43556-025-00264-9

**Published:** 2025-04-25

**Authors:** Yue Kong, Haokun Yang, Rong Nie, Xuxiang Zhang, Fan Zuo, Hongtao Zhang, Xin Nian

**Affiliations:** 1 Department of Endocrinology, The First Affiliated Hospital of Kunming Medical University, Kunming, China; 2 Kunming Medical University, Kunming, China; 3 Hangzhou CytoCan Biotech, Hangzhou, 310029 China

**Keywords:** Berberine, Complications, Natural product, Obesity, Pathophysiology, Therapies

## Abstract

Over the past few decades, obesity has transitioned from a localized health concern to a pressing global public health crisis affecting over 650 million adults globally, as documented by WHO epidemiological surveys. As a chronic metabolic disorder characterized by pathological adipose tissue expansion, chronic inflammation, and neuroendocrine dysregulation that disrupts systemic homeostasis and impairs physiological functions, obesity is rarely an isolated condition; rather, it is frequently complicated by severe comorbidities that collectively elevate mortality risks. Despite advances in nutritional science and public health initiatives, sustained weight management success rates and prevention in obesity remain limited, underscoring its recognition as a multifactorial disease influenced by genetic, environmental, and behavioral determinants. Notably, the escalating prevalence of obesity and its earlier onset in younger populations have intensified the urgency to develop novel therapeutic agents that simultaneously ensure efficacy and safety. This review aims to elucidate the pathophysiological mechanisms underlying obesity, analyze its major complications—including type 2 diabetes mellitus (T2DM), cardiovascular diseases (CVD), non-alcoholic fatty liver disease (NAFLD), obesity-related respiratory disorders, obesity-related nephropathy (ORN), musculoskeletal impairments, malignancies, and psychological comorbidities—and critically evaluate current anti-obesity strategies. Particular emphasis is placed on emerging pharmacological interventions, exemplified by plant-derived natural compounds such as berberine (BBR), with a focus on their molecular mechanisms, clinical efficacy, and therapeutic advantages. By integrating mechanistic insights with clinical evidence, this review seeks to provide innovative perspectives for developing safe, accessible, and effective obesity treatments.

## Introduction

As a chronic disease, Obesity characterized by excessive or abnormally distributed adipose tissue (AT) accumulation, is clinically defined by body mass index (BMI ≥ 30 kg/m^2^) and recognized as a multifactorial disorder driven by systemic energy imbalance [1–3]. Beyond caloric surplus, emerging evidence implicates interconnected etiological drivers, including obesogenic environments (e.g., hyperpalatable diets, sedentary technologies), gut dysbiosis, genetic predispositions, and epigenetic modifications, which collectively disrupt metabolic homeostasis [4]. Critically, obesity propagates a self-reinforcing cycle of complications—T2DM, CVD, NAFLD, respiratory diseases, ORN, malignancies, musculoskeletal disorders, and psychological comorbidities-contributing to elevated morbidity, mortality, and healthcare expenditures globally [5, 6].

Alarmingly, global obesity prevalence has surged over five decades, with projections indicating 1.9 billion affected adults by 2035 [7, 8]. In China, a nationwide cross-sectional research (n = 15.8 million) revealed 34.8% overweight (BMI 24–27.9 kg/m^2^) and 14.1% obese (BMI ≥ 28 kg/m^2^) individuals, with obesity-associated comorbidities disproportionately burdening this cohort (P < 0.001) [9]. This escalating epidemic underscores an urgent need for innovative therapeutic strategies, as current interventions, lifestyle modification, pharmacotherapy and bariatric surgery, face limitations: suboptimal efficacy (5–15% weight loss), adverse effects (e.g., gastrointestinal intolerance, surgical risks), and poor long-term adherence.

Against this backdrop, natural compounds like BBR, an isoquinoline alkaloid from Coptis chinensis, emerge as promising candidates. With a 70-year safety record in treating infectious diarrhea in China and preclinical evidence of multi-target anti-obesity actions (e.g., AMPK activation, gut microbiota modulation), BBR represents a paradigm shift toward accessible, low-cost therapies [10]. However, translational barriers persist, notably poor oral bioavailability (< 5%) and insufficient obesity-specific clinical validation.

This review synthesizes obesity’s pathophysiological axes, expounds the pathogenesis of common obesity complications, focuses on summarizing new anti-obesity drugs and targets, evaluates therapeutic gaps, and highlights BBR’s mechanistic novelty—including adipose browning and epigenetic regulation—while proposing formulation innovations (e.g., nanoparticle delivery, structural analogs) to bridge preclinical promise to clinical impact. By contextualizing BBR within the obesity therapeutic landscape, this work advances a roadmap for next-generation anti-obesity agents combining efficacy, safety, and scalability.

## Pathophysiology of obesity

Considering the substantial differences in how individuals respond to obesity treatment and the intricate etiology of obesity, a profound comprehension of the pathophysiological mechanisms underlying obesity is of utmost consequence for formulating reasonable, efficacious, and cost-efficient intervention strategies. Through a comprehensive review of the existing literature on the pathophysiology of obesity, the following is a summary of its pathophysiological mechanisms.

### The imbalance of energy homeostasis and metabolic adaptation

**Fig. 1:**
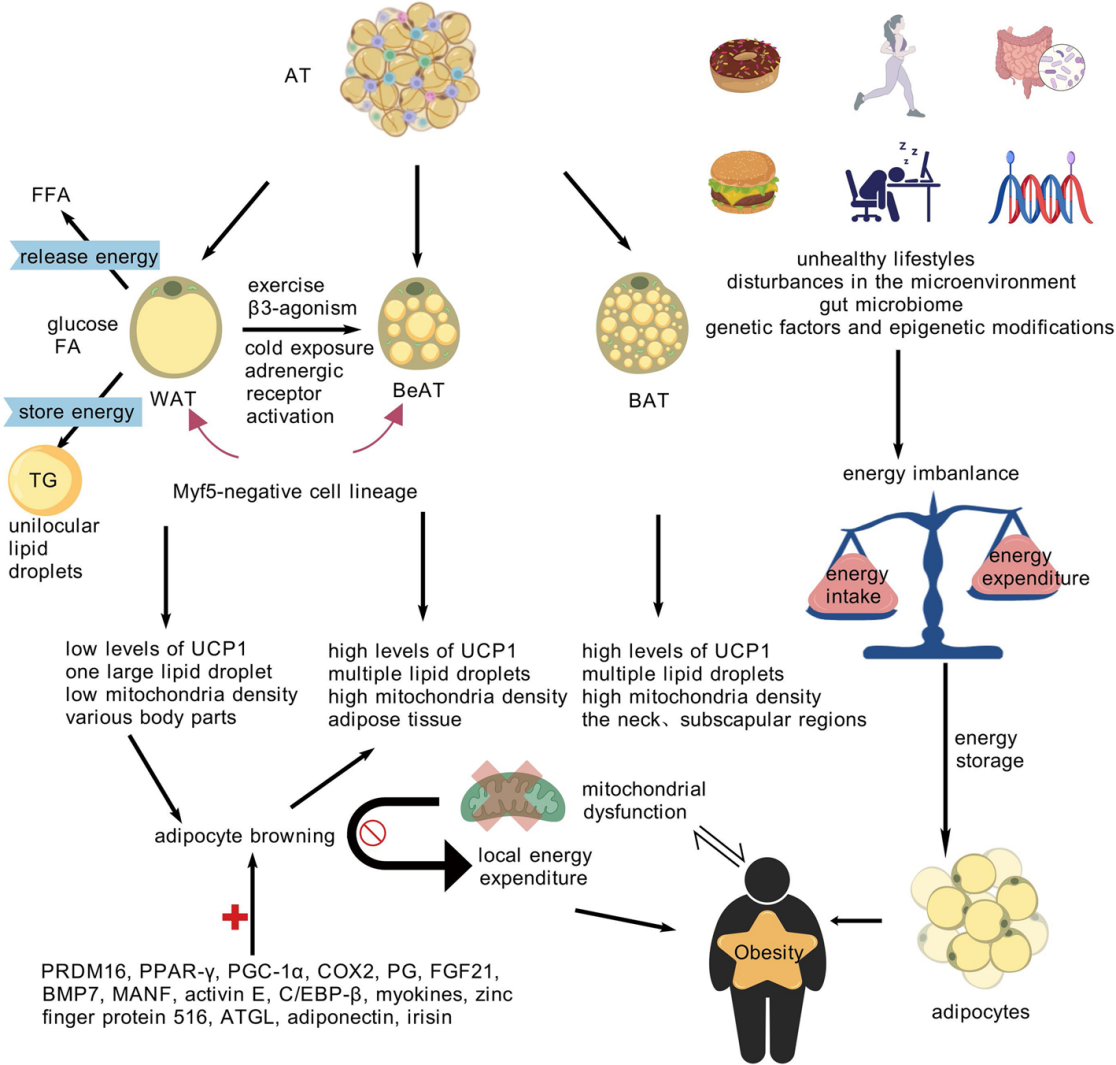
Energy Homeostasis and Metabolic Adaptation of Obesity. Obesity arises from disrupted energy homeostasis, characterized by excessive TG storage in WAT and impaired thermogenic capacity of BAT/BeAT. Mitochondrial dysfunction and suppressed WAT browning perpetuate metabolic inflexibility, driving IR and systemic metabolic disease. Interventions targeting adipose plasticity and mitochondrial health hold therapeutic potential. Figure 1 was created with BioGDP.com. AT, adipose tissue; WAT, white adipose tissue; BAT, brown adipose tissue; BeAT, beige adipose tissue; UCP1, uncoupling protein 1; FFA, free fatty acids; TG, triglycerides

The three primary components of energy homeostasis are energy intake, expenditure, and storage. Long-term energy storage occurs in adipocytes as intracellular droplets of triacylglycerol (TG). Adipocytes are mostly arranged in distinct AT depots where can be divided into five main categories: subcutaneous, visceral or intraperitoneal, pelvic and retroperitoneal, intra- or extra-pericardial and intramuscular [11, 12]. An energy imbalance is a hallmark of obesity (Fig. 1), where energy intake surpasses energy expenditure and the extra energy is retained in adipocytes [7].

AT predominantly exists in two primary forms: brown AT (BAT) and white AT (WAT), each of which has distinct physiological roles (Fig. 1). While WAT is dispersed throughout the body, BAT is predominantly located in the cervical and subscapular regionsis [13]. WAT primarily functions to store energy by converting glucose and fatty acids (FA) into triglycerides, which are housed in large unilocular lipid droplets. Subsequently, these are released as free fatty acids (FFA) within adipocytes [14]. In contrast, BAT is mitochondrial and essential for promoting energy expenditure and non-shivering thermogenesis [15, 16]. The thermogenic properties of BAT are largely due to its high mitochondrial density and the presence of uncoupling protein 1 (UCP1), which facilitates heat production by disrupting the normal process of oxidative phosphorylation [17].

Apart from WAT and BAT, another intermediate form between the WAT and BAT is beige adipose tissue (BeAT) (Fig. 1), which is frequently found inside WAT. Despite BeAT resembles BAT in morphology, it typically originate from a Myf5-negative cell lineage, akin to WAT. Beige adipocytes are adept at producing heat by separating lipid oxidation from ATP synthesis [13]. Research indicates that beige adipocytes can develop from specific preadipocyte populations [18] inside subcutaneous WAT or trans-differentiate pre-existing WAT [19, 20]. The process of WAT browning (Fig. 1) describes the process by which BeAT is generated within WAT. This transformation enhances energy expenditure, reduces the detrimental impacts of excessive WAT storage, and ultimately improves metabolic wellbeing [15, 21, 22].

The coordinated operation of the three types of AT guarantees an optimal metabolic state. This process involves a precisely coordinated structural and metabolic reorganization in response to physiological cues, enabling metabolic adaptability to satisfy the body’s requirements [23]. These metabolic and thermogenic reactions are mainly propelled by the distinctive features of the mitochondrial population. Mitochondrial malfunction disrupts the metabolic flexibility of adipocytes, contributing to metabolic disorders such as insulin resistance (IR), obesity and T2DM [24]. These metabolic changes initiate a vicious cycle that exerts a negative influence on the functionality of AT and undermines overall metabolic homeostasis [25]. According to Huang et al., the HFD group mice’s and obese individuals’ WAT browning processes were inhibited, which in turn inhibited local energy expenditure and exacerbation of obesity-related conditions [26].

### Hormonal regulation

**Fig. 2:**
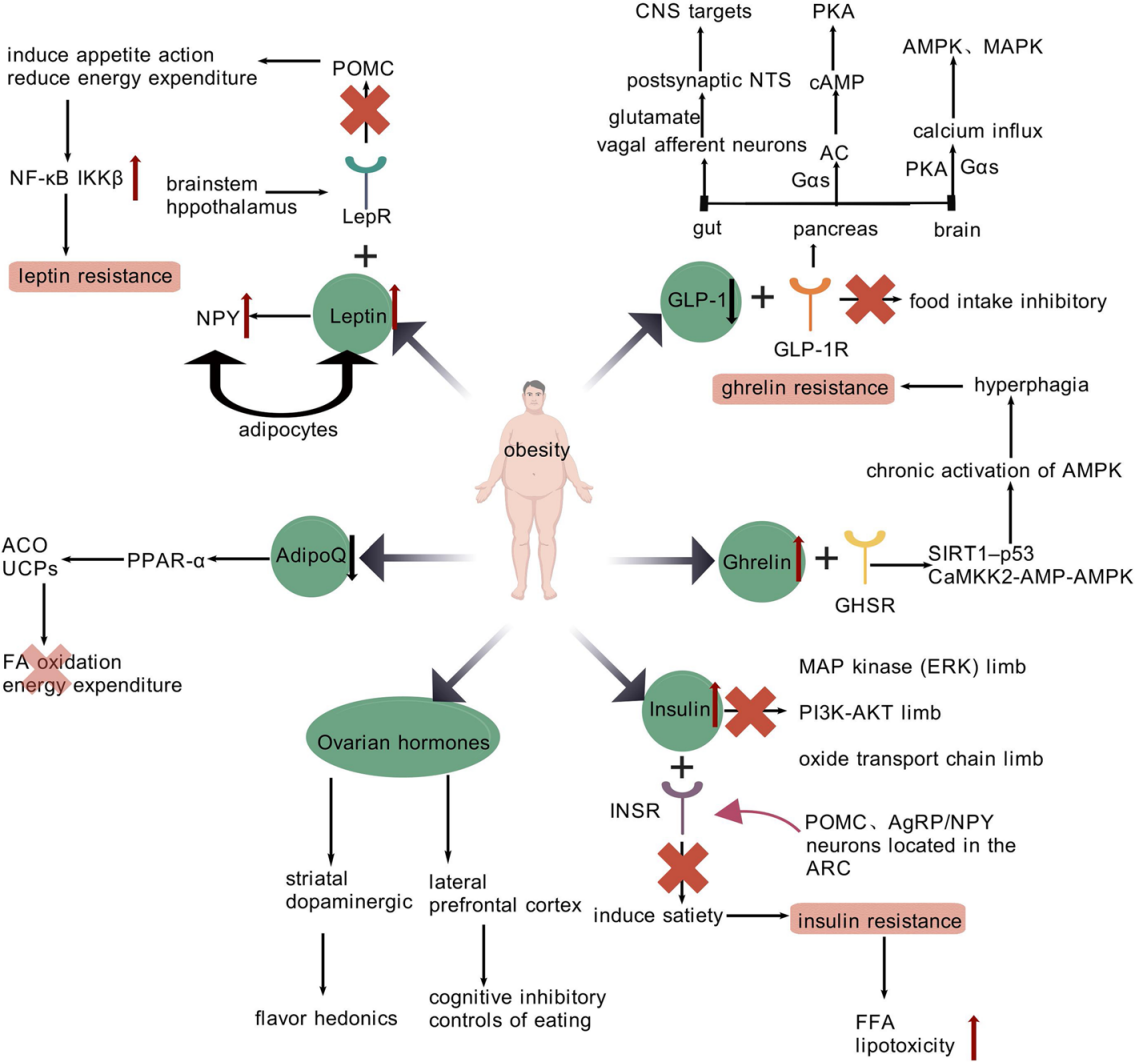
Hormonal Regulation Associated with Obesity. Obesity involves dysregulation of key hormones: Leptin resistance drives hyperphagia and energy imbalance. Insulin resistance promotes ectopic lipid accumulation and systemic metabolic dysfunction. Ghrelin suppression fails to counteract overeating. Adiponectin deficiency impairs lipid oxidation. GLP- 1 analogs offer therapeutic benefits by enhancing satiety. Ovarian hormones modulate hedonic and cognitive aspects of eating. Targeting leptin/insulin signaling, enhancing GLP- 1 activity, and restoring adiponectin levels may mitigate obesity-related metabolic disorders. Figure 2 was created with BioGDP.com. LepR, leptin receptor; POMC, pro-opiomelanocortin; ARC, arcuate nucleus; NPY, neuropeptide Y; NF-κB, nuclear factor-κB; IKKβ, NF-κB kinase-β; INSR, insulin receptor; AgRP/NPY, agouti-related peptide and neuropeptide Y; GHSR, growth hormone secretagogue receptor; SIRT1, sirtuin 1; CaMKK2, calcium/calmodulin-dependent protein kinase kinase 2; AMPK, AMP-activated protein kinase; ACO, acetyl CoA oxidase; UCPs, uncoupling proteins; GLP- 1, Glucagon-like peptide- 1; GLP- 1R, GLP- 1 receptor; Gαs, G protein α subunit; cAMP, cyclic AMP; PKA, protein kinase A; MAPK, mitogen-activated protein kinase; NTS, nucleus tractus solitarius

Adipocytes secrete a range of cytokines, including leptin, vastatin, interleukin- 6 (IL—6), adiponectin, tumor necrosis factor-alpha (TNF-α), resistin, angiotensinogen, aromatase, and adipsin. These bioactive molecules are integral to the regulation of appetite, satiety, and body fat content. When their normal regulatory mechanisms are disrupted, it can lead to IR associated with obesity as well as obesity itself, as illustrated in Fig. 2 [27, 28]. Gjermeni et al. claims that the primary factors regulating energy balance are insulin and leptin [29].

Leptin, encoded by the obese gene, mediates its biological functions through binding and activation of specific leptin receptor (LepR) following its production and release by adipocytes within WAT (Fig. 2) [30]. This adipocyte-derived hormone serves as a signaling molecule that communicates the body’s nutritional state, particularly during conditions of energy deficit. Consequently, physiological states characterized by caloric restriction or reduced adiposity demonstrate significant decreases in circulating leptin concentrations [31]. Leptin can influence appetite and calorie intake by binding to receptors that express pro-opiomelanocortin (POMC) in the brainstem, hypothalamus and arcuate nucleus (ARC) [32]. Mechanistically, leptin suppresses the activity of hypothalamic ARC neurons responsible for secreting neuropeptide Y (NPY), a potent orexigenic mediator that enhances hunger signals and reduces metabolic energy utilization, thereby promoting adipose accumulation [33]. HFD can trigger the activation of nuclear factor-κB (NF-κB) and its upstream regulatory factor, inhibitor of NF-κB kinase-β (IKKβ). This activation occurs by increasing endoplasmic reticulum (ER) stress within the hypothalamus. Such a series of events potentially results in the development of leptin resistance [34]. Leptin resistance manifests as diminished satiety signaling, hyperphagia, and progressive body mass accumulation, serving as key contributors to metabolic dysregulation in obesity [30]. The active free form of circulating leptin in individuals with obesity accounts for 85% of total leptin, inducing long-form leptin receptor (LepRb) desensitization through chronic overstimulation [35]. Although the actual quantity of leptin present in the cerebrospinal fluid (CSF) of overweight individuals might be greater compared to that of slender people, the efficacy of leptin’s transportation across the blood—brain barrier (BBB) (quantified by the ratio of CSF to plasma leptin) drops by up to 80% among the obese [36, 37]. Similarly, HFD rapidly activates astrocytes, causing inflammation and hyperleptinemia. In addition, a prolonged HFD further rouses astrocytes and promotes inflammation, which decreases the amount of leptin that reaches the brain [38, 39]. Based on these discoveries, it can be concluded that the transportation of leptin across the BBB is impaired in obese individuals.

The body can effectively handle the burden of dietary fat (such as triglycerides), protein, and carbohydrates thanks to the post-meal elevation in plasma insulin levels. Three signaling pathways, namely the mitogen-activated protein (MAP) kinase (extracellular signal-regulated kinase, ERK) pathway, the metabolic (phosphatidylinositol 3-kinase-protein kinase B, PI3K-AKT) pathway, and the oxidative transport chain pathway, are used by Williams et al. to summarize the underlying mechanisms [40]. As illustrated in Fig. 2, insulin-mediated satiation occurs via receptor activation within ARC nucleus POMC and agouti-related peptide and NPY (AgRP/NPY) neural circuits that govern energy homeostasis, nutrient partitioning, and glucose regulation [41]. Additionally, numerous researches indicated that prolonged overeating, temporary inactivity, sedentary behavior, and sleep deprivation all raised whole-body IR [42–44]. Because of enhanced lipolysis, hyperinsulinemia results in decreased development of AT while promoting lipolytic release of FFAs from triglyceride depots. However, a major cause of IR, ectopic lipid deposition and lipotoxicity are brought on by the excessive accumulation of FFAs in insulin-sensitive non-AT in obese individuals [45]. The pathophysiological progression of insulin resistance demonstrates bidirectional interactions with obesity (Fig. 2), constituting a fundamental mechanism underlying obesity-associated metabolic comorbidities [46].

Initially characterized as a growth hormone (GH)-releasing peptide, ghrelin (Fig. 2) has emerged as a pleiotropic regulator of energy balance, exhibiting inverse correlations with BMI and direct involvement in appetite modulation [47, 48]. In situations of positive energy balance, such as obesity, the expression of ghrelin is down-regulated. Conversely, in states of under-nutrition, like anorexia nervosa, its expression is up-regulated [49, 50]. In the ventromedial nucleus of the hypothalamus (VMH), ghrelin activates the cellular energy sensor AMP-activated protein kinase (AMPK) [51] via binding to growth hormone secretagogue receptor (GHSR) and stimulating the calcium/calmodulin-dependent protein kinase kinase 2 (CaMKK2)-AMPK axis [52] and hypothalamic sirtuin 1 (SIRT1)-p53 axis [53]. In both mice and humans, ghrelin-dependent hyperphagia and obesity are promoted by chronic AMPK activation [54]. Additionally, obese mice have lower levels of GHSR expression and ghrelin transport across the BBB, which results in decreased ghrelin sensitivity and may encourage hypothalamic ghrelin resistance [55, 56].

Through its autocrine activity, adiponectin (AdipoQ) (Fig. 2), an adipocyte-derived cytokine encoded by the chromosome's AdipoQ gene, aids in the development of adipocyte cells [57]. By significantly boosting PPAR-α expression and activity, which leads to the up-regulation of acetyl CoA oxidase (ACO) and uncoupling proteins (UCPs), AdipoQ stimulates FA oxidation and energy expenditure (Fig. 2) [58]. AdipoQ demonstrates proportional relationships with insulin sensitivity that become attenuated in obese states, as evidenced by clinical biomarker studies [59, 60]. According to Singh’s research, AT in obese individuals exhibits decreased AdipoQ secretion due to compromised leptin signaling and elevated caveolin- 1 expression [61].

The gut, brainstem, and endocrine pancreas all express Glucagon-like peptide- 1 (GLP- 1), which binds to GLP- 1 receptor (GLP- 1R) to regulate energy balance (Fig. 2) [62]. Through G protein α subunit (Gαs), GLP- 1R stimulates adenylate cyclase and raises cyclic AMP (cAMP) levels in the pancreas. This, in turn, initiates protein kinase A (PKA)-dependent intracellular signaling pathways, which ultimately trigger the release of insulin and induce genetic modifications [63–66]. Through Gαs and PKA, GLP- 1R stimulates mitogen-activated protein kinase (MAPK) and AMPK and improves calcium influx through VGCCs in the brain [67]. When nutrients pass through the gut, they cause the release of GLP- 1and then interact with GLP- 1Rs on the vagus nerve to activate the vagal afferent neurons. These neurons release glutamate, which excites postsynaptic nucleus tractus solitarius (NTS) neurons with specific phenotypes. The axons of NTS neurons project monosynaptically to anatomically distributed targets in the central nervous system (CNS), influencing the excitability of neurons in identified nuclei. This process leads to the release of GLP- 1 in the brain and contributes to the inhibitory regulation of food intake [68, 69]. Experimental models utilizing transgenic reporter mice revealed that liraglutide potentiates GABAergic neurotransmission, directly stimulating POMC neurons while indirectly suppressing AgRP/NPY activity through GABA receptor-mediated inhibition [70]. Furthermore, GLP- 1 lowers the rate of gastric emptying (GE) by activating myenteric neurons and vagal afferent nerves, it promotes satiety, reduces calorie intake, and has anorexigenic effects. GLP- 1 is linked to the mechanism that promotes meal termination for cholecystokinin (CCK) [71]. By cleaving an X-Pro or X-Ala dipeptide, dipeptidyl-peptidase IV (DPP-IV) renders GLP- 1 inactive [72]. Clinical investigations confirm these mechanisms, demonstrating that GLP- 1 infusion in non-obese fasting subjects significantly attenuates hunger perception while enhancing satiety signaling [73]. A multicenter RCT evaluating daily 3 mg liraglutide administration in overweight/obese participants (BMI > 27 kg/m^2^ with comorbidities or > 30 kg/m^2^) reported 8.4 kg mean weight reduction over 56 weeks, with 33.1% achieving > 10% body weight loss [74].

Ovarian hormones critically modulate the endocrine mechanisms underlying obesity (Fig. 2). Research by Leeners et al. demonstrates that cyclic variations in ovarian hormones alter feeding behavior by modifying two neural pathways: cognitive inhibitory control of appetite mediated by the lateral prefrontal cortex, and dopamine-dependent reward processing in the striatum governing food palatability. Their findings further reveal that elevated estrogen levels during the pre-ovulatory phase suppress caloric intake through dual mechanisms: enhancing the satiety-inducing effects of gastrointestinal peptide cholecystokinin (CCK) while simultaneously reducing hedonic responses to sweet-tasting foods in the follicular phase [75].

### Neural control

**Fig. 3:**
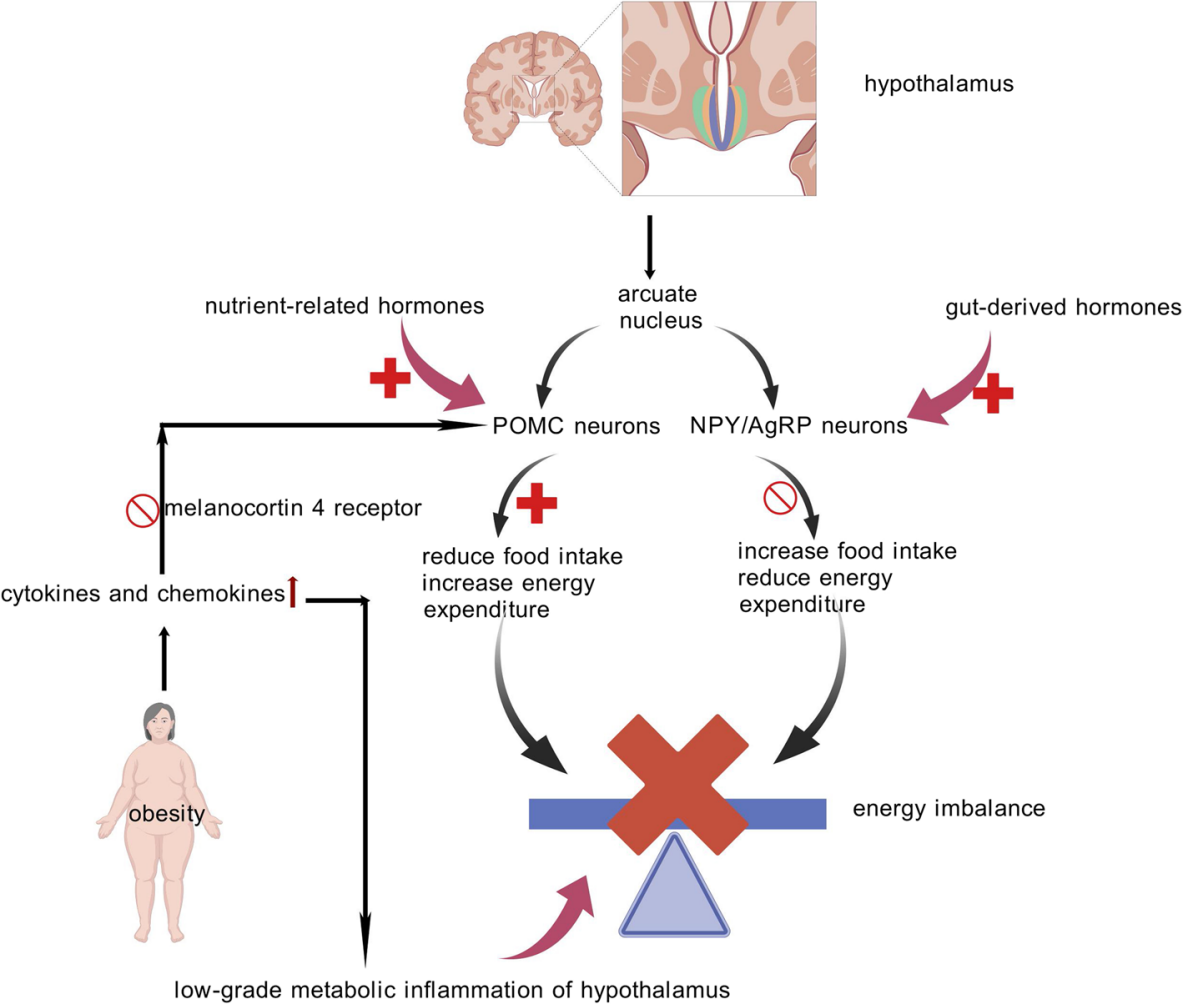
The Neural Control of Obesity. The hypothalamic arcuate nucleus (ARC) regulates feeding behavior and energy homeostasis through two opposing neuronal populations: anorexigenic POMC neurons (reducing food intake via MC4R activation and increasing energy expenditure) and orexigenic NPY/AgRP neurons (promoting hunger and suppressing energy use). Chronic overnutrition (e.g., HFD, leptin deficiency) disrupts ARC plasticity, impairing homeostatic regulation through mechanisms like leptin signaling defects, ER stress, and metabolic inflammation. This inflammation alters neuropeptide secretion, desensitizes energy-balance neurons, and exacerbates dysregulation of appetite and metabolism. Figure 3 was created with BioGDP.com

A crucial regulatory center for feeding behavior and whole-body energy homeostasis, the ARC of the hypothalamus (Fig. 3) relays information between the periphery nervous system (PNS) and the CNS [76]. The ARC contains two functionally antagonistic neuronal groups, POMC and NPY/AgRP neurons, which collaboratively regulate systemic energy homeostasis, and integrate central and peripheral inputs [77].

POMC-derived melanocortin peptides exert catabolic effects via stimulation of melanocortin 4 receptor (MC4R)-positive neural circuits, resulting in appetite suppression and enhanced metabolic rate. Conversely, by counteracting these effects, NPY/AgRP neurons demonstrate anabolic functions through MC4R-dependent antagonism, promoting caloric conservation via reduced thermogenesis while stimulating feeding motivation [78, 79]. Notably under energy surplus conditions, circulating satiety factors predominantly activate the POMC neuronal network to inhibit ingestive behavior and increase energy dissipation. Conversely, during negative energy balance, gastrointestinal-derived orexigenic signals preferentially engage NPY/AgRP neurons to amplify food-seeking drives while suppressing catabolic processes [80–82].

It is noteworthy that persistent food excess (HFD or leptin insufficiency) inhibits neurogenesis, which in turn impairs hypothalamic homeostatic regulation and dynamical plasticity [83]. Leptin receptor signaling pathway defects, ER stress, and decreased leptin transport across the BBB are some possible explanations [84–86]. Accumulating evidence indicates that diet-induced obesity correlates with chronic neuroinflammatory responses within the ARC of obese animal models [87]. Notably, hypothalamic inflammation disrupts POMC neuron functionality through dual mechanisms: cytokine-mediated alteration of neuropeptide secretion profiles, and impaired neuronal plasticity that compromises adaptive energy regulation. This pathological cascade establishes a self-perforcing cycle where disrupted neurotransmission exacerbates inflammatory signaling, ultimately leading to dysregulated appetite control and metabolic inflexibility [88, 89].

### Inflammation and immune responses

**Fig. 4:**
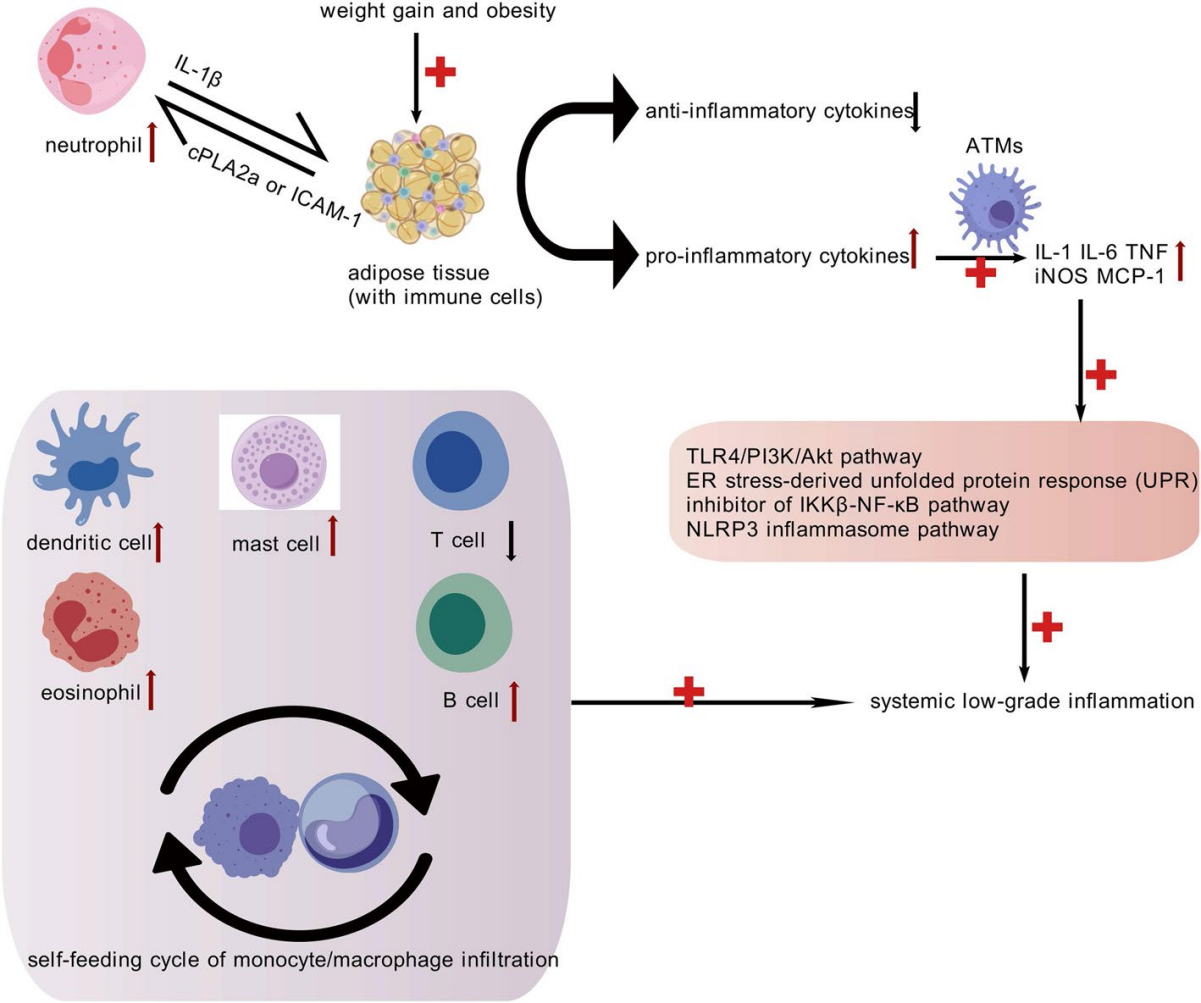
The Inflammation and Immune Responses of Obesity. Obesity induces AT inflammation via pro-inflammatory cytokines from dysfunctional adipocytes and immune cells, causing IR and systemic low-grade inflammation. Metabolic pathways like TLR4/PI3 K/Akt, ER stress, and NLRP3 inflammasome are involved. Immune dysregulation further exacerbates IR and chronic inflammation. Figure 4 was created with BioGDP.com. TLR4/PI3 K/Akt pathway, toll-like receptor 4/phosphatidylinositol- 3'- kinase/Protein kinase B pathway; NLRP3, nucleotide-binding and oligomerization domain (NOD) leucine-rich repeat family pyrin domain-containing 3; ATMs, adipose tissue macrophages; iNOS, inducible NO synthase; MCP- 1, monocyte chemoattractant protein- 1; cPLA2α, cytosolic phospholipase-A2α; ICAM- 1, endothelial cell adhesion molecule 1

AT is essential to the pathophysiology of obesity and has a major impact on physiological and pathological processes, such as immunological responses and inflammation [90]. Both pro- and anti-inflammatory cytokines are secreted by AT’s immune cells. Pro-inflammatory cytokines promote IR and cause detrimental lipid metabolism in peripheral tissues, whereas anti-inflammatory cytokines attempt to preserve insulin sensitivity [91, 92]. The progression of weight gain and subsequent obesity induces a phenotypic shift in WAT. This shift results in the formation of dysfunctional and inflammatory adipocytes, accompanied by the recruitment of immune cells into the stromal vascular compartment [93]. Inflammatory and defective adipocytes secrete pro-inflammatory cytokines both locally and systemically. This secretion results in systemic low-grade inflammation (Fig. 4).

Among the key pathways implicated in adipose tissue (AT) inflammation are the toll-like receptor 4 (TLR4)/phosphatidylinositol- 3’-kinase (PI3 K)/protein kinase B (Akt) pathway, the ER stress-induced unfolded protein response (UPR), and the IKKβ-NF-κB inhibitory pathway are three metabolic pathways that are significant in the development of AT inflammation [94]. Moreover, an important modulator of metabolic inflammation is the nucleotide-binding and oligomerization domain (NOD) leucine-rich repeat family pyrin domain-containing 3 (NLRP3) inflammasome pathway [94]. Supporting above-mentioned conclusions, De et al. discovered that the consumption of a HFD upregulates the expression of pro-inflammatory cytokines, including IL- 1, IL- 6 and TNF, in the hypothalamus, thereby triggering inflammatory cascades [95]. Furthermore, inflammation and leptin resistance related to obesity have been linked to ER stress [96, 97]. Through a rise in reactive oxygen species (ROS) mediated by FFAs, an HFD in mice causes ER stress and persistent inflammation in WAT [98].

It is well recognized that obesity affects immunological function, just like other forms of malnutrition (Fig. 4). Weisberg et al. have indicated that activated adipose tissue macrophages (ATMs) are the primary sources of pro-inflammatory mediators. These include TNF-α, inducible nitric oxide synthase (iNOS), monocyte chemoattractant protein- 1 (MCP- 1), and IL- 6 [99, 100]. There is additional evidence suggesting that obesity perpetuates low-grade chronic inflammation in WAT by triggering a self-perpetuating cycle of monocyte/macrophage infiltration [101, 102].

Additionally, it seems that additional immune cells are involved in AT inflammation. According to recent reports, bidirectional interaction between adipocytes and neutrophils can trigger WAT inflammation by activating the endothelial cell adhesion protein ICAM- 1 or cytosolic phospholipase-A2α (cPLA2a), which then leads to the production of IL- 1β [103, 104]. According to certain research, obese people have lower levels of regulatory T cells (Tregs), which may lead to long-term WAT inflammation and IR [105–107]. Additionally, obesity has been linked to increased B cell, mast cell, dendritic cell, and eosinophil activity, which activates T cells and AMTs to cause IR [108–111].

### Genetics and epigenetics

**Fig. 5:**
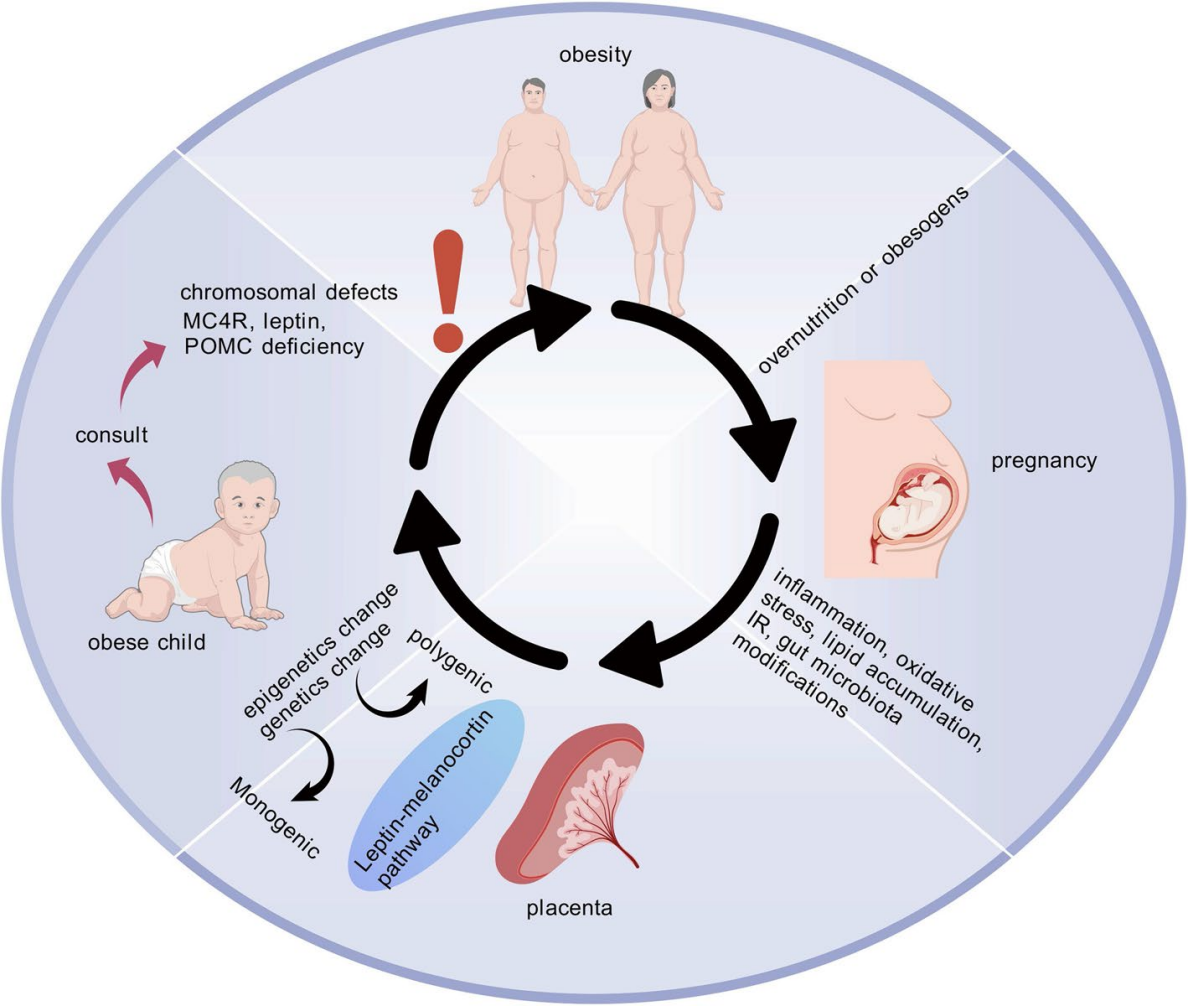
Genetic and Epigenetic Mechanisms of Obesity. Genetic and epigenetic mechanisms critically shape obesity susceptibility. Maternal obesity, particularly in obesogenic or hyper-nutritional environments during pregnancy, induces metabolic dysregulation characterized by placental exposure to inflammation, oxidative stress, lipid accumulation, insulin resistance, and gut microbiota alterations. These pathological conditions drive epigenetic modifications and genetic mutations, thereby predisposing offspring to childhood obesity. This mechanistic cascade underscores the necessity of early clinical screening to identify at-risk populations and implement targeted preventive or therapeutic interventions. Figure 5 was created with BioGDP.com. MC4R, melanocortin 4 receptor; IR, insulin resistance

In addition, it is well recognized that hereditary variables are widely acknowledged as crucial determinants of an individual’s susceptibility to obesity (Fig. 5) [112]. Twin and familial research indicates that hereditary influences account for 40 to 70 percent of the variability in human obesity [113]. Obesity genetic causes can be broadly categorized as either polygenic or monogenic mutations. The leptin-melanocortin pathway is the primary cause of monogenic obesity, and numerous genes, including AgRP, PYY (orexogenic) or MC4R, interfere with the appetite and weight regulation system [114]. Numerous genes work together to create polygenic obesity [115]. Neurodevelopmental abnormalities and other organ/system anomalies can lead to syndromic obesity, a severe form of obesity that may be brought by alterations in a wider chromosomal region that encompasses multiple genes [116].

Based on Martins’ research, those who have severe obesity with an onset prior to the age of two are advised to seek advice from experts in obesity medicine (Fig. 5). They should also contemplate undergoing screening for MC4R deficiency, POMC deficiency, and leptin deficiency [117]. Similarly, obesity additionally arises from chromosomal abnormalities, such as deletion of 17p11.2 (Smith Magenis syndrome), 11p13 (WAGR syndrome), 9q34 (Kleefstra syndrome), 6q16 (PWS-like syndrome), 2q37 (brachydactyly mental retardation syndrome), and 1p36 (monosomy 1p36 syndrome) [118].

Epigenetic alterations, such as modifications in DNA methylation, histone tails, and microRNAs (miRNAs), have emerged as crucial means for comprehensively analyzing the widespread prevalence of the obesity epidemic (Fig. 5). The most significant epigenetic mechanism for controlling gene expression seems to be DNA methylation. Houde’s study [119] uncovered a correlation between LDL-C levels and the DNA methylation status of the AdipoQ gene and leptin-encoding gene. Maternal metabolic health can also shape the DNA methylation profile of leptin at birth, thereby influencing the metabolic reprogramming associated with obesity [120]. In a similar vein, reduced methylation levels in the regions of insulin-like growth factor 2 (IGF2) have been associated with paternal obesity [121]. G protein-coupled receptor 75 (GPCR 75), functioning as a ciliary protein expressed in the brain, is predominantly found in the primary cilia of hypothalamic neurons and associated with a lower BMI. The ciliary positioning of GPCR 75 is essential for its functionality and role in controlling the formation of fat tissue [122, 123].

Severe obesity is strongly tied to mutations in various GPCRs that govern neuro-endocrine processes, including those in the stimulatory Gαs and particular adenylate cyclases, such as ADCY3, which regulate feeding, satiety, adipogenesis, and fat accumulation [124–126]. Additionally, environmental factors such as obesogens, alterations in gut microbiota composition, and dietary imbalances can contribute to weight gain and metabolic dysregulation through epigenetic mechanisms [127].

### Gut microbiome dysbiosis

**Fig. 6:**
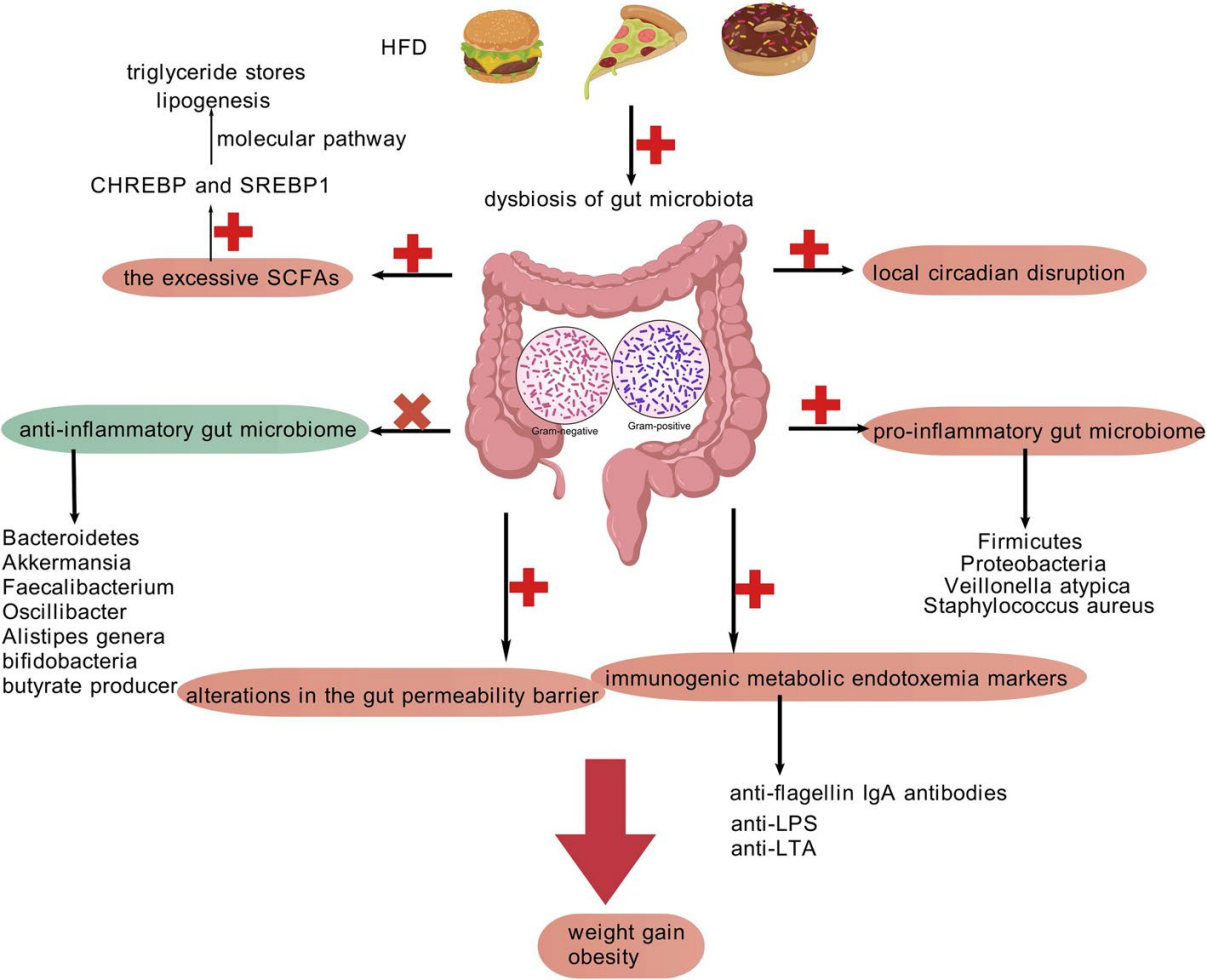
The Gut Microbiota Dysbiosis of Obesity. Gut microbiota dysbiosis contributes to obesity via multiple mechanisms: altered microbial composition, circadian disruption, SCFA-mediated lipogenesis, pathogenic strain activity, and compromised intestinal barrier integrity. Therapeutic strategies targeting microbial balance, barrier restoration, or specific taxa may mitigate obesity-related metabolic disorders. Figure 6 was created with BioGDP.com. HFD, high fat diet; LPS, lipopolysaccaride; SCFAs, Short-Chain Fatty Acids; CHREBP, carbohydrate responsive element-binding protein; SREBP1, sterol regulatory element-binding transcription factor- 1

There has been discussion over the role that early microbial dysbiosis plays in the development of metabolic diseases and obesity. Based on this, Ebert examined cause and effect in mice, and their research clearly shows that early microbial deprivation did not affect adiposity but instead caused IR and altered the expression of liver genes linked to glucose metabolism in mice [128]. Furthermore, a accumulating body of research indicates that metabolic disorders like obesity and T2DM have been associated to prolong intestinal dysbiosis (Fig. 6) [129]. To elucidate the causal relationship between human gut microbiota and obesity development, researchers conducted fecal microbiota transplantation (FMT) from adult humans to germ-free (GF) murine models. The experimental outcomes demonstrated that recipient mice colonized with microbiota from obese donors exhibited significant increases in adiposity, body mass, and metabolic dysfunction biomarkers [130].

Circadian rhythmicity represents a crucial modulator of gut microbial homeostasis, with its disruption leading to substantial alterations in intestinal microbial composition [131, 132]. Numerous studies have demonstrated that disruptions in the gut microbiota, stemming either from antibiotic-induced depletion or long-term HFD intake, are capable of triggering local circadian rhythm disruptions that contribute to weight gain [132–135].

The Firmicutes/Bacteroidetes ratio (F/B ratio), a key indicator of microbial community structure, has been associated with multiple pathological conditions, including metabolic disorders [136]. Empirical evidence supports the observation of increased F/B ratios in individuals with obesity [137, 138]. Furthermore, epidemiological investigations have identified reduced *Bifidobacterium* abundance and elevated *Staphylococcus aureus* colonization in specific populations, particularly among obese pregnant women and overweight pediatric cases [139–141]. In line with Gaber’s data, anti-lipopolysaccharide (anti-LPS), anti-lipoteichoic acid (anti-LTA), and anti-flagellin IgA antibodies are among the immunogenic markers of metabolic endotoxemia linked to visceral AT (VAT) in postmenopausal women. Additionally, there is an increase in pro-inflammatory components of the gut microbiome, such as Proteobacteria (including *Escherichia coli*, *Shigella spp.*, and *Klebsiella spp.*) and Veillonella atypica [142]. When given to mice fed a HFD without germs, Enterobacter cloacae strain B29, which has been isolated from the Enterobacteriaceae, has been demonstrated to induce obesity [143]. Faecalibacterium prausnitzii, a butyrate-producing bacterium renowned for its anti-inflammatory qualities, has been observed to be reduced in individuals with diabetes who are morbidly obese [144]. In particular, compared to people of normal weight, obese individuals have been observed to have significantly lower levels of the bacterial species Akkermansia, Oscillibacter, and Alistipes [145].

Furthermore, introducing A. muciniphila into mice improves intestinal barrier function and decreases body weight growth, fat mass formation, and low-grade inflammation [146]. Likewise, through a molecular pathway, an elevation in the concentration of Short-Chain Fatty Acids (SCFAs) in the plasma of obese individuals can activate carbohydrate responsive element-binding protein (CHREBP) and sterol regulatory element-binding transcription factor- 1 (SREBP1), which in turn can drive lipogenesis, increase triglyceride storage and then obesity [147, 148]. Nicholson’s research team has established correlations between intestinal barrier dysfunction, localized inflammatory responses, and microbial community imbalance [149].

## Comorbidities associated with obesity

**Fig. 7:**
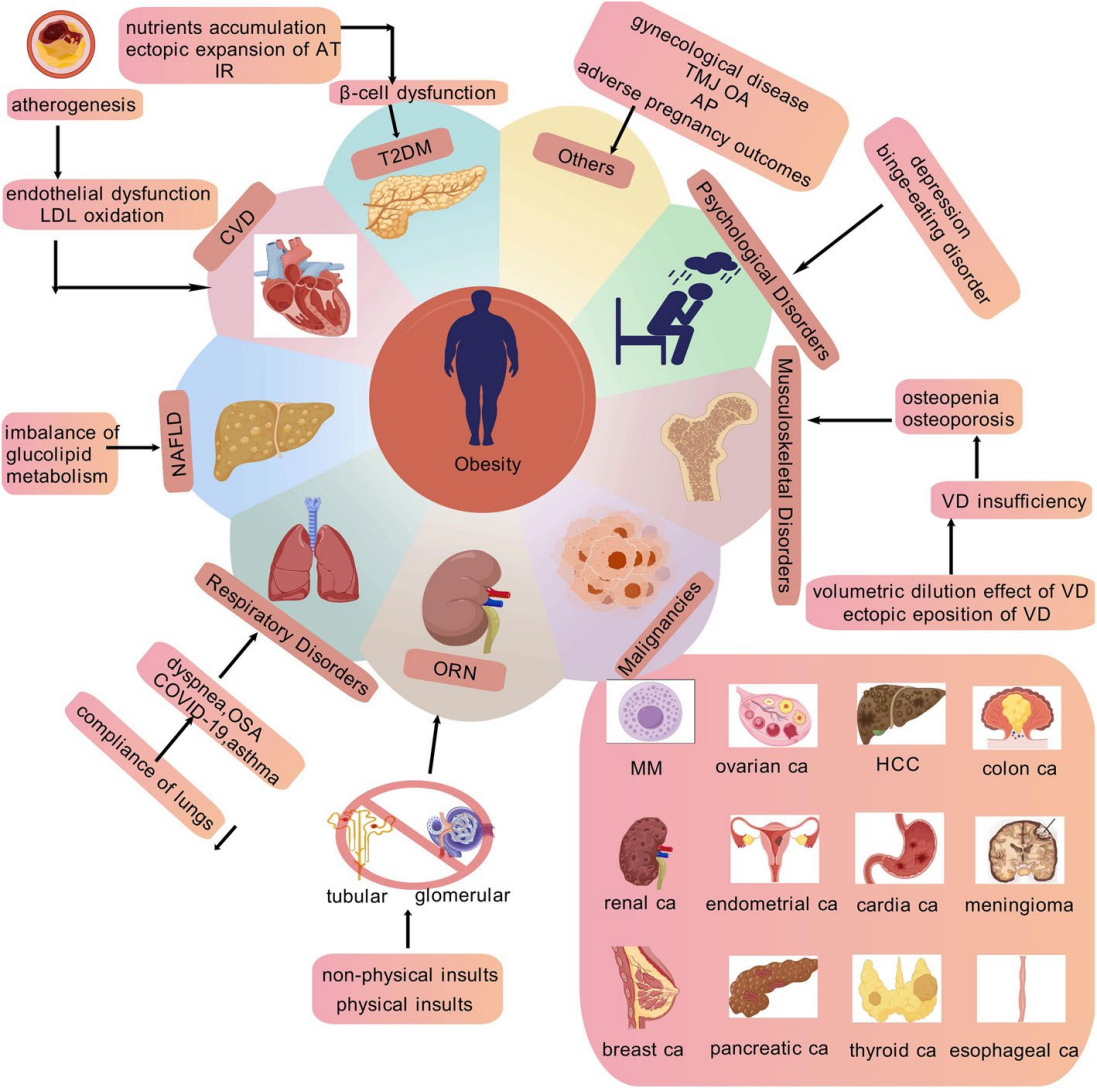
The Complications of Obesity. The complications of obesity mainly include T2DM, CVD, NAFLD, respiratory disorders, ORN, musculoskeletal disorders, cancers, psychological comorbidities and others. Figure 7 was created with BioGDP.com. T2DM, type 2 diabetes mellitus; CVD, cardiovascular diseases; NAFLD, non-alcoholic fatty liver disease; ORN, obesity-related nephropathy; APCs, adipocyte precursor cells; LDL, Low Density Lipoprotein; CAD, coronary artery disease; SCD, sudden cardiac death; MASH, metabolic dysfunction-associated steatohepatitis; HCC, hepatocellular carcinoma; OSA, obstructive sleep apnea; VEGF-B, vascular endothelial growth factor-B; ROS, reactive oxygen species; ER, endoplasmic reticulum; VD, Vitamin D; MM, multiple myeloma; Ca, cancer; POCS, polycystic ovary syndrome; TMJ OA, Temporomandibular joint osteoarthritis; AP, acute pancreatitis

Furthermore, concentrating only on obesity is insufficient. A chronic and recurring condition, obesity either causes or exacerbates other diseases. Obesity-related comorbidities are associated with higher morbidity, disability, and mortality. Therefore, this review will deeply understand the pathogenesis of related common complications from the perspective of obesity (Fig. 7), in order to identify and intervene the complications of obesity early, so as to effectively prevent the occurrence of more serious complications and improve the clinical treatment effect of obesity.

### Type 2 diabetes mellitus (T2DM)

According to a World Health Organization (WHO) prediction in 2009, 439 million people worldwide will have diabetes by 2030 [150]. Upstream diseases of T2DM include pre-obesity/obesity, metabolic dysfunction linked to steatotic liver disease, and dyslipidemia, which typically manifests prior to T2DM [151]. One of the main causes of IR is obesity [152]. The abnormal expansion of AT in non-adipose sites (ectopic expansion) and the over-accumulation of specific nutrients and metabolites,

once obesogenic factors amplify genetic susceptibilities, disrupt the metabolic equilibrium through IR, impaired autophagy and the microbiome-gut-brain axis. Consequently, systemic inflammation is triggered, which further exacerbates the dysregulation of immunometabolism. which frequently results in early β-cell malfunction, accelerates the deterioration of β-cell function and gradually raises blood glucose levels, ultimately leading to T2DM [153].

Similarly, the build-up of senescent cells in the subcutaneous AT and the functional decline of adipocyte precursor cells (APCs) in obese people both contribute to the development of T2DM [154–156]. In accordance with Desiderio et al., the- 1317 CpG at the PANDAR promoter became hypo-methylated in obesity, progressively inducing senescence in APCs in subjects with obesity, which worsens along the progression toward T2DM [157]. However, in certain people with IR, T2DM can also develop inversely before obesity, leading to raised insulin levels and increased hepatic glucose production-the actual causes of obesity [158].

### Cardiovascular disease (CVD)

Based on the data from the China Health and Retirement Longitudinal Study (CHARLS) that screened 7703 individuals, Jiang et al. suggest that sarcopenic obesity, and potentially other related conditions, are positively correlated with the development of CVD [159]. Previous evidences suggested that obesity is linked to numerous kinds of CVD, including stroke [160], venous thromboembolic disease [161], pulmonary hypertension [162], atherosclerotic CAD [163], heart failure (HF) [164], arrhythmias especially sudden cardiac death (SCD) [165] and atrial fibrillation (AF) [166]. The risk of ischemic and hemorrhagic strokes increases by 4% and 6% for every unit increase in BMI, respectively [167].

Early atherosclerotic alterations are accelerated by obesity via several mechanisms, such as inflammation and IR [168]. Obesity-induced inflammation raises the risk of LDL oxidation, which in turn promotes atherogenesis [169]. The development of atherosclerosis is also fundamentally influenced by endothelial dysfunction in obesity, which is principally brought on by diminished NO bioavailability in the context of inflammation and oxidative stress [170]. BMI in the overweight and obese ranges was linked to an elevated risk of CAD, according to a meta-analysis of nearly 300,000 people with 18,000 CAD occurrences [171]. For every 10-kg increment in body weight, there is a 12% elevation in the risk of CAD, along with a 3-mmHg increase in systolic blood pressure and a 2.3-mmHg increase in diastolic blood pressure [172].

In addition, as demonstrated by growing research, non-obstructive coronary artery disease (NOCAD) is also quite common in ischemia or chest pain (CP) and has a significant financial impact [173–175]. In 814 patients with angiographically confirmed NOCAD, the results showed that obesity was independently linked to the occurrence of NOCAD-related CP and that those who were obese had a higher prevalence of NOCAD-related CP (77.6% vs 67%, *P* < 0.001) and more frequent NOCAD-related CP (angina frequency composite score, 74.9 vs 78.3, *P* = 0.02) than those who were not obese [176]. However, when comparing obese patients undergoing bariatric surgery to those who did not get surgical intervention, Karason et al. found that the former had improved CP [177]. Adipocytes in obese persons specifically prevent endothelial-mediated vasodilatation of the coronary microvasculature, which results in coronary heart disease (CMD) and an oxygen-supply demand mismatch, according to the mechanism of the association between obesity and NOCAD-related CP [178, 179].

Simultaneously, obesity heightens the risk of myocardial dysfunction and HF through multiple mechanisms. These include hemodynamic changes, neurohormonal activation, the endocrine and paracrine effects of AT, lipotoxicity, and ectopic fat deposition [164]. However, Zhou et al. indicated that the protective effects of obesity persists in people with chronic HF (CHF) irrespective of metabolic status [180], the phenomenon called obesity paradox. The risk of AF was directly related to BMI, increasing by 4.7% (95% CI: 3.4 to 6.1, *P* < 0.0001) for each kilogram per square meter [181]. Atrial fibrillation is caused by a combination of left atrial dilatation and dysfunction, elevated epicardial fat, adipocyte infiltration of the myocardium, and fibrosis [182–184].

### Non-alcoholic fatty liver disease (NAFLD)

NAFLD is defined as steatosis is not caused by alcohol, drugs, or viral-induced steatosis and with ≥ 5% fat infiltration in imaging or histology [185]. In line with the growing prevalence of obesity, the prevalence of NAFLD is also rapidly rising [186]. Obesity can have an impact on whole-body glucose and lipid metabolism, which exacerbates the overproduction or excessive uptake in the liver and lipid droplet buildup in the hepatic parenchyma [187, 188]. It is the excessive deposit of fat within the liver cells that result in inflammation, fibrosis and cirrhosis, Momo's studies established that obese subjects significantly tend to have higher liver enzymes like ALT and AST than non-obese adults(serum ALT: 37.14 ± 15.18U/L vs 21.92 ± 5.10 U/L, serum AST: 41.15 ± 15.24U/L vs 25.01 ± 6.65U/L) [189].

Histologically, metabolic dysfunction-associated steatohepatitis (MASH) is characterized by the co-existence of steatosis, inflammation, and hepatocyte injury (ballooning) [185]. According to Schmidt-Christensen et al., mice fed obesogenic diets develop MASH, steatosis, and hepatocyte ballooning more quickly [190]. While the majority of NAFLD patients simply show steatosis and no further development, some will experience negative effects from their liver disease, including cirrhosis, MASH, and hepatocellular carcinoma (HCC) [191].

### Obesity-related respiratory disorders

It is well—established that obesity is associated with the secretion of adipokines and pro-inflammatory factors. These substances have the potential to intensify inflammatory states [192]. Obesity is closely linked to a variety of respiratory diseases and has an impact on the prognoses of acute respiratory distress syndrome (ARDS) and chronic obstructive pulmonary disease (COPD) [193]. Obesity has significant effects on respiratory function by reducing compliance of the lungs and chest wal and resting lung volumes, producing airway narrowing, closure and airway dysanapsis, increasing respiratory system resistance, which cause asthma and dyspnea, wheeze and airway hyper-responsiveness [194].

Sleep-disordered breathing, a highly prevalent condition in obese patients, is characterized by the collapse of the upper airway during sleep. The main pathophysiological mechanisms of obstructive sleep apnea (OSA) include chronic intermittent hypoxia, sleep fragmentation and inflammatory activation [195]. The results of Sands’s study, obesity is the largest risk factor for OSA at the population level (11–21 times higher than non-obesity) [196]. Obesity is linked to both increased collapsibility and increased loop gain presumably through increased tongue fat and decreased lung volume, which raises the chance of the OSA [197, 198].

Everyone is aware that asthma is a long-term inflammatory condition. According to a cross-sectional study involved 11,137 participants from NHANES 2011–2018, higher VAT is linked to a higher risk of developing asthma, especially in older people and women [199]. Research findings demonstrated that the VAT mass in asthma patients was 529 g, which was notably higher than the 455 g in the non-asthma group. In three distinct models (the unadjusted model, the model adjusted for demographic factors, and the fully adjusted model), for every 200-g increase in VAT, the risk of asthma increased by 10.4%, 20.8%, and 20.3% respectively.

A life-course Mendelian randomization study was carried out with the aim of exploring the causal impacts of early life adiposity on the COVID- 19 susceptibility and severity. They found that childhood BMI and obesity were positively correlated with COVID- 19 risk and severity in adulthood, and revealed strong evidence of a genetically predicted effect of childhood obesity on COVID- 19 hospitalization [200].

### Obesity-related nephropathy (ORN)

Epidemiological investigations by Wang and colleagues demonstrate that obesity-associated renal pathologies account for approximately one-quarter to one-third (24–33%) of chronic kidney disease cases documented in American clinical populations [201]. Substantiating this global health concern, a comprehensive retrospective cohort analysis conducted by Hu’s research team at Zhengzhou University examined 34,630 primary renal biopsy specimens, revealing a significant temporal progression in obesity-related glomerulopathy prevalence from 0.86% (2009) to 1.65% (2018) [202]. Aforementioned statistical trends indicate a progressive annual elevation in ORN diagnosis rates across diverse demographics. This correlation receives further validation from population-scale research involving 320,000 subjects, which established a positive association between incremental BMI elevations and corresponding escalations in end-stage renal disease (ESRD) risk profiles [203].

The pathophysiological mechanisms underlying ORN involve dual injury modalities affecting renal microarchitecture. Mechanical stressors manifest through altered glomerular hemodynamics, visceral adipose-induced renal compression, and podocyte deformation from sustained mechanical tension. Concurrently, metabolic disturbances include RAAS overactivation, bile acid homeostasis disruption, insulin resistance, lipid-induced cellular toxicity, and chronic inflammatory cascades [204]. To be specific, by means of the vascular endothelial growth factor-B (VEGF-B) signaling pathway, mitochondrial damage and the ensuing increase in IR, ROS production, and ER stress, a HFD has been shown to encourage lipid accumulation in mice, ultimately leading to renal impairment [205, 206]. Additionally, a two-sample Mendelian randomization research conducted in European populations confirmed that renal function impairment, which is fueled by adverse obesity, is linked to genetically high BMI [207].

Proteinuria, glomerulomegaly, increasing glomerulosclerosis, and decreased kidney function are clinical characteristics of ORN [208]. However, due to the lack of specificity in clinical parameters and histopathological features, ORN is easily confused with other causes of chronic kidney disease. Emerging diagnostic approaches emphasize the detection of tubular injury biomarkers, with urinary kidney injury molecule- 1 (KIM- 1), cystatin C, N-acetyl-beta-D-glucosaminidase (NAG) enzyme activity, and neutrophil gelatinase-associated lipocalin (NGAL) protein concentrations showing particular promise for early ORN identification in clinical urinalysis [209, 210]. Meanwhile, multiple studies have established that obesity and hyperuricemic nephropathy (HN) have connections [211, 212].

### Musculoskeletal impairments

The preservation of bone tissue and the homeostasis of the minerals calcium and phosphorus depend on vitamin D (VD). A cross-sectional study aimed to evaluate the VD levels among 1,210 obese individuals in Southern Morocco, their results (adequate: 5.3%, insufficiency: 18%, moderate-deficiency: 52.5%, severe-deficiency: 24.2%) support the hypothesis that obesity is associated with low VD levels [213]. Moreover, volumetric dilution effect of VD is the most probable mechanism for the reduction of serum VD concentration in obese patients [214]. In particular, 25-(OH)D is mostly found in the liver, muscle, fat, and serum, all of which are elevated in obesity [215]. Wortsman et al. suggest that VD insufficiency associated with obesity may result from reduced bioavailability of VD, as it tends to accumulate in AT, thereby limiting its availability from both cutaneous synthesis and dietary intake [216].

Consequently, obese individuals may need higher initial doses of VD supplementation to achieve serum 25-(OH)D levels comparable to those of individuals with normal body weight. Devlin’s findings further support this by demonstrating that obesity negatively impacts bone health, contributing to conditions such as osteopenia and osteoporosis [217]. Numerous variables, including hyperinflammation, genetics, microbial dysbiosis, hypermetabolism, and local alterations in the bone marrow environment, are involved in the mechanisms of obesity-related bone dysregulation [218]. Additionally, a longitudinal study conducted over four years in a middle-aged and elderly Chinese population revealed that the co-occurrence of dynapenia and abdominal obesity significantly elevated the risk of developing arthritis in women (RR: 1.39, 95% CI: 1.01–1.93) [219].

### Malignancies

All obesity-related malignancies are estimated to have a general population-attributable percentage of 11.9% in men and 13.1% in women [220]. Obesity has been identified as a significant risk factor for malignancies in at least 13 anatomical regions, including the endometrium, esophagus, kidneys, pancreas, liver, gastric cardia, meninges, multiple myeloma, colorectum, breast, ovaries, gallbladder, and thyroid [221].

Both clinically severe estrogen-independent type II and estrogen-dependent type I endometrial carcinoma (EC) are independently associated with obesity [222]. Risks for EC are increased in obese women and high visceral abdominal fat volume (VAV)% independently predicts reduced EC survival [223]. Notably, Schlottmann et al. highlight a concurrent rise in the prevalence of overweight and obesity with the incidence rates of esophageal adenocarcinoma (EAC) [224]. Furthermore, a comprehensive meta-analysis encompassing 24 studies and over 8 million participants revealed that BMI is positively correlated with an elevated risk of renal cell carcinoma (RCC) in both males (RR 1.05 for every 1 kg/m^2^ increase) and women (RR 1.06 for every 1 kg/m^2^ increase) [225].

Obesity also significantly increases the risk of pancreatic cancer, obesity-induced pancreatic inflammation and desmoplasia, which contributed to pancreatic ductal adenocarcinoma (PDAC) progression and chemotherapy resistance [226]. HCC has been increasingly associated with metabolic diseases such as the metabolic syndrome, which often co-occur with NAFLD or NASH [227]. Epidemiological studies consistently indicate that elevated BMI and obesity are significant risk factors for the development of cardia cancer [228]. A US population-based study, combined with a multi-institutional cohort analysis, revealed that obese males are more prone to meningiomas at the skull base compared to other locations. Additionally, patients undergoing meningioma resection are more likely to be obese than those with other intracranial tumors [229].

Obesity has been positively linked to both the mortality and incidence of multiple myeloma (MM) in both prospective cohort and case–control studies [230–232]. According to the findings of a Mendelian randomization research, men are more likely to develop colorectal cancer (CRC) if their BMI is more extensive, whereas women are more likely to develop CRC if their waist-to-hip ratio (WHR) is higher [233]. In addition, patients who are overweight or obese have a 1.2–1.4 times higher risk of developing postmenopausal breast cancer [234]. Multiple research studies confirm that obesity is closely associated with the risk of developing papillary thyroid carcinoma (PTC) [235, 236]. Li et al. propose that obesity may facilitate the progression of PTC by suppressing adiponectin expression [237]. Meanwhile, obesity also can increase the risk of invasion (OR = 1.395) and lymph node metastasis (OR = 1.387) [238]. Similarly, obesity increases the risk of benign tumors. A case–control study investigating the relationship between visceral fat and uterine fibroids found that higher levels of body fat, particularly abdominal visceral fat, significantly raise the risk of developing uterine fibroids [239].

### Psychological comorbidities

Obesity and depression frequently co-occur and exacerbate each other [240, 241]. A bidirectional relationship has been established between obesity and depression, wherein obesity or being overweight increases the likelihood of depressive symptoms, and conversely, depression elevates the risk of obesity or overweight [242–244]. The physical condition and weight issues associated with obesity ultimately increase the likelihood of developing depression by decreasing self-esteem, social isolation and dissatisfaction with body image. Meanwhile, the depression often include emotional instability, poor in appetite and reduced energy expenditure, which can lead to disrupted eating behaviors, decreased physical activity and ultimately result in weight gain and obesity. But the relationship between obesity and depression exist individual differences, not all individuals with obesity will experience symptoms of depression, and not all individuals with depression will develop obesity [245].

Individuals with obesity often experience disordered eating patterns, with binge-eating disorder being the most common among this population [246]. Compared with regular-weight patients and those without an Eating Disorders (EDs), obese patients seem to express peculiarities regarding the expression of some emotional processes, including impulsivity, aggression and anger [247]. Metabolic and vascular dysfunction of obesity, including inflammation, IR and leptin resistance, have been considered as the key risks to depression and anxiety development [248]. According to Kalarchian et al., social anxiety disorder is the most prevalent anxiety disorder among candidates for bariatric surgery, affecting 9% of patients [249].

### Other comorbidities

A population-based cohort study aimed to quantify the contribution of overweight and obesity to various adverse pregnancy outcomes in Swedish females. As estimated by Population attributable fractions, a significant percentage of unfavorable pregnancy outcomes were caused by overweight and obesity: gestational diabetes (52.1%), large-for-gestational age (36.9%), pre-eclampsia (26.5%), low Apgar score (14.7%), infant mortality (12.7%), severe maternal near-miss event (8.5%) and preterm birth (5.0%) in the total study population [250]. Furthermore, numerous researches have consistently demonstrated that obesity is associated with higher rates of miscarriage, unfavorable perinatal outcomes in assisted reproductive technology (ART), and reduced rates of implantation, pregnancy, and live delivery [251, 252].

In women of reproductive age, obesity also raises the risk of diseases including polycystic ovary syndrome (PCOS), irregular menstruation, decreased ovarian reserve, ovulatory dysfunction, subfecundity, and increased incidence of preeclampsia, stillbirth, and miscarriage [253, 254]. A UK population-based cohort supports that women who gain/change weight between pregnancies may increase the incidence of overweight/obesity (≥ 85 th centile) and obesity (≥ 95 th centile) in second children [255].

Li et al. unveiled that obesity and the related metabolic changes were important influencing factors for Temporomandibular joint osteoarthritis (TMJ OA) [256]. Simultaneously, multiple studies have confirmed the association between obesity and acute pancreatitis (AP). There is a significant association between severe AP and VAT in a single-centre prospective study (VAT area: severe AP: 141.01 ± 33.75 cm^2^ vs moderate AP: 115.11 ± 29.85 cm^2^), incorporating VAT into one of the prognostic indices for AP needs to be further explored [257].

## Interventions of obesity

**Fig. 8:**
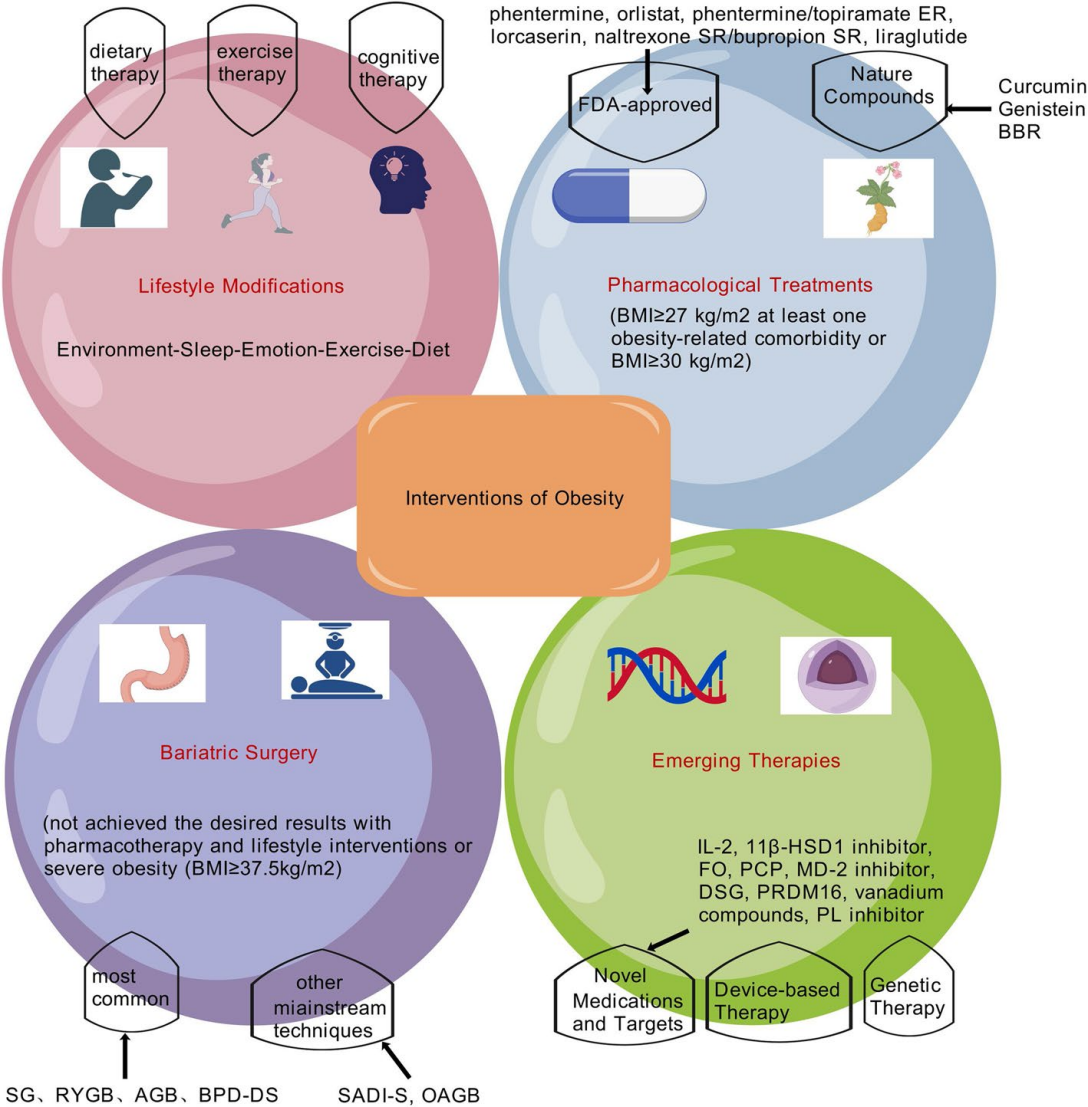
The Interventions of Obesity. The primarily treatment interventions for obesity are summarized, which principally include lifestyle intervention, pharmacotherapy, bariatric surgery, and emerging therapies. Figure 8 was created with BioGDP.com. ER, extended release; SR, sustained release; SG, Sleeve gastrectomy; RYGB, Roux-en-Y gastric bypass; AGB, adjustable gastric banding; BPD-DS, biliopancreatic diversion with duodenal switch; OAGB, One anastomosis gastric bypass; SADI-S, single-anastomosis duodenal ileostomy with sleeve gastrectomy; BBR, Berberine; 11β-HSD1, 11β-hydroxysteroid dehydrogenase type 1; FO, fish oil; PCP, Penthorum chinense Pursh; MD- 2, Myeloid differentiation factor 2; DSG, Diosgenin; PL, pancreatic lipases

Current evidence-based guidelines recommend lifestyle interventions, pharmacotherapy, and bariatric surgery (e.g., sleeve gastrectomy, gastric bypass) as primary obesity treatments. However, these approaches face significant limitations, including weight regain and safety concerns even with gold-standard therapies [6]. Persistent adverse effects—such as cardiovascular risks, gastrointestinal complications, and metabolic disturbances—associated with existing anti-obesity drugs have heightened the demand for safer, sustainable alternatives. Consequently, natural products and plant-derived bioactive compounds are increasingly investigated for their therapeutic potential. The mechanisms and efficacy of major obesity interventions are systematically categorized in Fig. 8 and Table 1.

**Table 1:** Current and Emerging Interventions for Obesity Management

Treatment Method	Specific Measures	Clinical Efficacy	Advantages	Disadvantages	Ref
**a** Lifestyle modifications					
Dietary Therapy	ADF; Ramadan diet; Anti-inflammatory diet	A 24-h ADF regimen prevented HFD-induced obesity and precocious puberty; Participants demonstrated an average weight reduction of 2.3 ± 0.99 kg during the 29-day Ramadan month; An 8-week anti-inflammatory diet intervention resulted in significant weight loss (5–8.6 kg) compared to initial weight	Non-invasive, improves metabolic health, low cost	High dropout rates, weight regain common,35–50% non-responders	[260–262]
Exercise Therapy	Redistributing screen time; AE, HIIT	Substitution of 1-h screen time with reading time was inversely associated with obesity risk; Overweight female participants exhibited BMI reductions of 5.81% and 5.06% following AE and HIIT, respectively			[264, 266]
Cognitive Therapy	Nutritious balanced diet	Body weight reduction of 5.7 ± 5.3 kg at the one-year follow-up			[269]
**b** Pharmacological Treatments					
FDA-Approved	Phentermine	Weight loss relative to placebo ranging from approximately 6%	Rapid short-term appetite suppression, effective for initial weight loss	Dry mouth, constipation, insomnia, arrhythmias, increased blood pressure	[487]
	Orlistat	Weight loss relative to placebo ranging from approximately 3%	Non-central acting, reduces dietary fat absorption	Oily stools/spotting, flatus, fecal urgency, tubulointerstitial nephritis	[488]
	Phentermine/topiramate ER	Weight loss relative to placebo ranging from approximately 8.6–9.3%	Dual-action: appetite suppression and satiety enhancement, greater weight loss	Oral clefts, paresthesia, dizziness, insomnia, dysguesia,constipation, kidney injury, dry mouth	[489]
	Lorcaserin	weight loss relative to placebo ranging from approximately 3-3.6%	a selective serotonin 2C (5-HT2C) receptor agonist	Headache, dizziness, fatigue, nausea, dry mouth, constipation, cough	[490]
	Naltrexone/bupropion SR	Weight loss relative to placebo ranging from approximately 3.3–4.8%	Dual-action, reduces appetite and improves mood	Nausea, headache, constipation, vomiting, dry mouth, kidney injury,dizziness	[491]
	Liraglutide	Weight loss relative to placebo ranging from approximately 4% to 6.2% with diabetes compared with 6.1% to 17.4% without diabetes	Improves insulin sensitivity, lipid profile, cardiometabolic and renal benefits, sustained weight loss, pediatric use	High costs, nausea, diarrhea, pancreatic safety, thyroid cancer, gallbladder events, injection-site, allergic reactions	[287]
Natural Compounds	Curcumin	A greater BMI reduction compared to placebo (25.94 kg/m2 vs 29.34 kg/m2) in obese patients with T2DM after 12-month RCT	Anti-inflammatory and anti-oxidant effects, metabolic benefits, safety	Low oral bioavailability, limited long-term clinical validation, transient gastrointestinal side effects	[317]
	Genistein	Genistein attenuated body weight gain in gonadectomized mice with diet-induced obesity	Mitigates obesity-related metabolic dysfunctions, particularly NAFLD and glucose metabolism	Insufficient clinical trial data, potential estrogenic effects, unclear optimal dosing for metabolic benefits	[321]
	Berberine	BBR significantly reduced multiple adiposity parameters including body weight, BMI, body fat percentage, visceral fat percentage, and diastolic blood pressure	Multi-mechanistic anti-obesity effects, cardiometabolic benefits, effective and minimal side effects	Nausea, diarrhea, constipation, low oral bioavailability	[327, 333, 335, 337, 339, 339, 349]
**c** Bariatric Surgery					
	SG, RYGB, AGB, BPD-DS, SADI-S, OAGB	Comparative analysis of bariatric procedures revealed distinct total weight loss (TWL) percentages: AGB (36.29%), RYGB (31.59%), and SG (21.07%) during the first postoperative year	Long-term weight reduction, resolution of comorbidities, prolonged survival	Perceived invasiveness, high cost, nutritional deficiencies(e.g., VD), surgical risks(e.g., weight regain, DVT, pulmonary embolism, ulcers, gastroesophageal reflux disease dumping syndrome, apnoea)	[406–408, 410]
**d** Emerging Therapies					
Medications and Targets	IL- 2; 11β-HSD1 inhibitors; Marine FO; PCP; MD- 2 inhibitors; DSG; PRDM16; Vanadium compounds; PL inhibitors	Low-dose IL- 2 administration exhibited metabolic regulatory potential; 11β-HSD1 inhibitor ameliorated HFD-induced metabolic dysregulation; Marine FO exerted anti-obesity effects through weight reduction, BMI improvement, and modulation of serum lipids, leptin and TNF-α; PCP supplementation alleviated hyperglycemia and promoted weight loss; MD- 2 inhibitors attenuated obesity-related cognitive impairment; DSG suppressed ectopic lipid accumulation; PRDM16 modulated obesity and diabetes pathogenesis; Vanadium compounds notably attenuating adipose tissue accumulation and inflammation; PL inhibitors reduced adiposity in HFD-fed mice via enzymatic competition	Novel mechanisms, non-surgical options	Limited clinical data, engraftment instability, regulatory hurdles, immature technology, high cost	[438, 443, 449, 456, 458, 460, 466–472]
Device-based Therapies	Mobile smart devices-based health interventions, medical device based on PG, localized delivery system	Device-based therapies significantly improved obesity-related parameters including body weight, IR, cholesterol levels, and inflammatory markers			[474–476]
Genetic Therapies	AAV, miRNAs, Lep, MC4R and POMC gene, based on ADMSCs, FMTs	Genetic therapies ameliorated obesity-associated comorbidities: metabolic dysfunction, cellular senescence, osteoarthritis progression, oxidative DNA damage, and epigenetic hypermethylation			[477–479, 481, 483, 484]

This table summarizes the clinical efficacy, advantages, and limitations of established therapeutic approaches (lifestyle modification, pharmacotherapy, bariatric surgery) and investigational therapies discussed in this review

*ADF *alternate-day fasting, *AE *aerobic exercise, *HIIT *high-intensity interval training, *FDA *Food and Drug Administration, *ER *extended release, *SR *sustained release, *NAFLD *non-alcoholic fatty liver disease, *SG *Sleeve gastrectomy, *RYGB *Roux-en-Y gastric bypass, *AGB *adjustable gastric banding, *BPD-DS *biliopancreatic diversion with duodenal switch, *OAGB *One anastomosis gastric bypass, *SADI-S *single-anastomosis duodenal ileostomy with sleeve gastrectomy, *VD *vitamin D, *DVT *deep vein thrombosis, *11β-HSD1 *11β-hydroxysteroid dehydrogenase type 1, *FO *fish oil, *PCP *Penthorum chinense Pursh, *MD- 2 *Myeloid differentiation factor 2, *DSG *Diosgenin, *PRDM16 *PR domain-containing 16, *PL *pancreatic lipases, *PG *polyglucosamine polymers, *AAV *adeno-associated virus, *Lep *leptin, *LepR *leptin receptor, *MC4R *melanocortin 4 receptor, *POMC *pro-opiomelanocortin, *ADMSCs *adipose-derived mesenchymal stem cells, *FMTs *fecal microbiota transplants

### Lifestyle modifications

As the initial treatment for weight management and cardiovascular risk mitigation in obesity, comprehensive lifestyle modification (encompassing dietary regulation, physical activity optimization, and behavioral adaptation) forms the cornerstone of anti-obesity therapeutic intervention [258].

Contemporary research emphasizes the critical importance of nutritional modifications in modulating lipid parameters and pro-inflammatory mediators, given the pathophysiological relation between adiposity-related chronic low-grade inflammation and elevated cardiovascular risk [259]. According to Ullah’s research, sex hormone and growth hormone (GH) levels were lowered by alternate-day fasting (ADF), which resulted in slower growth and postponed puberty. Although precocious puberty and obesity brought on by an HFD were avoided by ADF, more clinical research is required to verify its safety [260]. AS one of the intermittent fasting practices, Ramadan fasting (fasted for an average of 14–15 h daily from dawn to sunset during the 29-day Ramadan month) induced weight loss (average weight loss of 2.3 ± 0.99 kg), modified gut microbiota (F/B ratio, Firmicutes phylum et al. significant decreases, Bacteroidetes and Proteobacteria phyla et al. significant increases), and improved blood lipid profile [261]. Recent investigations demonstrate that adopting anti-inflammatory dietary regimens, particularly those integrating Mediterranean nutritional principles with national dietary guidelines (TÜBER- 2016), effectively reduces both BMI and systemic inflammation through iso-caloric meal plans achieving negative dietary inflammatory indices (− 3.38 in females vs. − 3.53 in males) [262].

Certainly, most obese patients are difficult to control their weight through dietary intervention alone and usually need to be supplemented with exercise therapy. Adolescents'unhealthy food behaviors and overweight/obesity were strongly correlated with snacking while watching TV, according to a longitudinal study [263]. A longitudinal investigation spanning three years revealed that extended screen exposure duration exhibited strong positive correlation with adiposity indices (p < 0.01). Notably, strategic reallocation of screen time to purposeful physical/social activities (including structured exercise, interpersonal interactions, cognitive tasks, and restorative sleep) significantly attenuates obesity progression [264]. Porri’s assessment indicates that poor sleep hygiene can considerably contribute to weight growth and the worsening of metabolic diseases connected to pediatric obesity, but more thorough research is required [265]. Mechanistic studies by exercise physiologists further elucidate that structured exercise regimens, whether aerobic exercise (AE) or high-intensity interval training (HIIT), enhance circulating pentraxin- 3 (PTX3) concentrations (a cardioprotective inflammatory modulator) while favorably modifying lipoprotein profiles. Clinical trials document 5.81% and 5.06% BMI reductions respectively in overweight females following supervised training protocols [266].

While many individuals consider dieting to be the effective norm for weight loss, the reality is far more complex. Frequent intermittent dieting appears to be effective initially, but it can cause weight regain in people whether they are overweight/obese or not [267, 268]. Therefore, in addition to dietary intervention and exercise intervention, behavioral therapy for obese patients should also incorporate an essential component of cognitive therapy (Fig. 8). Significantly, it is necessary to alter the incorrect perception of diet control. Dietary intervention for obese patients does not merely mean dieting and abstaining from carbohydrate intake; instead, it involves adopting a healthy and nutritious balanced diet achieved through a reasonable nutritional ratio. Innovative digital health solutions hold particular promise in resource-limited settings. The EMPOWER initiative exemplifies this through its tripartite intervention model combining virtual nutritional education, personalized lifestyle coaching, and cloud-based progress tracking. Preliminary results show that, in the rural participant cohorts, there is a body weight reduction of 6.2% ± 6.0% (5.7 ± 5.3 kg) at the one-year follow-up [269].

In summary, lifestyle modifications may be a practical strategy to prevent obesity. However, lifestyle interventions are limited by poor compliance and efficacy. Nevertheless, clinical observations reveal notable interindividual variability, with 35–50% of patients failing to achieve clinically meaningful weight loss (≥ 5% baseline reduction) despite intensive behavioral protocols spanning 4–6 months [270, 271]. The fact that most people who do lose weight eventually gain it back is even more concerning [272]. In a similar vein, clinical and epidemiological investigations have revealed that minority of obese individuals are unwilling or unable to maintain long-term lifestyle changes [273].

All in all, lifestyle intervention requires early and comprehensive strategies, for example, the ‘magic polypill’ covering ‘Environment-Sleep-Emotion-Exercise-Diet [E(e)SEEDi]’, and long-term persistence to achieve ideal efficacy [274–276].

### Pharmacological treatments

#### Current anti-obesity medications

Current clinical guidelines specify pharmacological eligibility criteria for anti-obesity therapeutics, targeting individuals presenting with BMI values ≥ 27 kg/m^2^ with concurrent metabolic comorbidities (including T2DM, cardiovascular disorders, dyslipidemia, or SA) or those with BMI ≥ 30 kg/m^2^ irrespective of comorbidities. A growing number of medications have been authorized in recent years to treat obesity. The Food and Drug Administration (FDA)-endorsed pharmacopeia for chronic weight management currently comprises six principal agents: phentermine, orlistat, phentermine/topiramate extended release (ER) (combined GABAergic/glutamatergic agent), lorcaserin, naltrexone SR/bupropion sustained release (SR) (opioid-dopaminergic combination), and liraglutide [277].

Phentermine worked by either preventing norepinephrine from being reabsorbed or by promoting its release. The sole FDA-licensed anti-obesity drug that does not exert action in the brain is orlistat, a gastric lipase inhibitor that was approved in 1999 [278]. Topiramate is a gamma-aminobutyric acid agonist. Lorcaserin is a selective agonist of serotonin 2 C (5-HT2 C) receptor. Nltrexone is a non-selective antagonist of opioid receptor. Bupropion inhibits the transporters of norepinephrine and dopamine. Significant weight loss and cardiometabolic advantages are provided by new weight loss treatments, such as GLP- 1R agonists (GLP- 1 RAs), dual glucose-dependent insulinotropic polypeptide (GIP), and triple GIP, GLP- 1, and glucagon receptor agonists [279].

Mechanistically, GLP- 1 RAs exert pleiotropic effects through pancreatic β-cell preservation (enhancing proliferation while suppressing apoptosis), glucose-dependent insulinotropic/glucagonostatic regulation, and gastrointestinal motility modulation-collectively contributing to improved glycemic control and attenuated postprandial lipidemia [280–282]. Through GLP- 1Rs in the hypothalamus, GLP- 1 also decreases appetite, food intake, and promotes satiety [283]. Additionally, GLP- 1R signaling inhibits hepatocyte de novo lipogenesis and β-oxidation, reverses cholesterol transport, lowers the liver's hepatic TG content (HTGC) and VLDL-TG production rate, and modifies important liver enzymes involved in lipid metabolism [284]. GLP- 1 RAs may preserve free leptin levels by targeting areas in the hindbrain, simultaneously delay in gastric emptying and induce satiety [285].

GLP- 1 RAs include liraglutide (brand name,Victoza, Novo Nordisk, Copenhagen, Denmark), semaglutide (brand name, Wegovy, Novo Nordisk, Copenhagen, Denmark) and tirzepatide (brand name, Zepbound, Eli Lilly, Indianapolis, IN, USA), which are approved and marketed as weight-loss drugs. Semaglutide and liraglutide have now been approved in the US and Europe to treat obesity in children as young as 12 years of age [286]. The first GLP- 1 RAs to receive a license for long-term weight control was ligarglutide. In 2021, semaglutide—the next generation of GLP- 1 RAs—was authorized at weekly doses of up to 2.4 mg for the treatment of chronic obesity-related weight loss [278]. Systematic analysis by Jensterle et al. demonstrated mean differential weight reduction of 4.0–6.2% (vs placebo) when adjunctive to lifestyle interventions in diabetic patients, contrasting with 6.1–17.4% efficacy in non-diabetic populations through GLP- 1 RAs [287]. According to Ansari et al., GLP- 1 RAs have been further shown to help lower cardiovascular disease risk factors like blood pressure and lipid profile in addition to aiding in weight loss [288]. GLP- 1 RAs shown long-term beneficial effects on cardiovascular health, renal outcomes and adverse events in obese people in Huang’s extensive observational trial, which is consistent with the above conclusions [289].

GLP- 1 RAs can be used as a single treatment, in conjunction with other hormone-based drugs, or engineered as a dual or triple receptor agonist. Finding GLP- 1 RAs that additionally target either the glucagon receptor, the GIP receptor, or both has advanced the field [290]. In large quantities recent therapeutic advancements reveal superior efficacy profiles for novel agents: Tirzepatide (dual GIP/GLP- 1 receptor co-agonist) achieved 20.9% weight reduction at 15 mg dosing over 72 weeks [291], while retatrutide (triple GIP/GLP- 1/glucagon receptor agonist) demonstrated unprecedented 24.2% weight loss at 12 mg over 48 weeks in phase III trials [292].

Sodium-glucose cotransporter- 2 inhibitor (SGLT2i) and dipeptidyl peptidase- 4 inhibitors (DPP- 4is) have been demonstrated to improve blood pressure, lipid profiles, body weight, and endothelial function [293, 294]. Emerging evidence elucidates adiposity regulation through DPP- 4is, modulating WAT mass and thermogenic pathways via PPAR-α upregulation/UCP3 induction in skeletal muscle, coupled with BAT activation through GLP- 1/MC- 4 signaling cross-talk [295]. DPP- 4i further enhances β3-adrenergic signaling via ERK pathway suppression, potentiating UCP1-mediated thermogenesis in BAT and inguinal WAT (iWAT) depots to avoid obesity [296].

Concurrently, SGLT2i exhibit pleiotropic anti-inflammatory properties, suppressing NLRP3 inflammasome activity and pro-inflammatory cytokines (TNF-α, IL- 1β, IL- 6, IL- 18) in preclinical models [297, 298]. Clinically, SGLT2i demonstrates nephroprotective effects in obese T2DM patients, decelerating chronic kidney disease (CKD) progression irrespective of glycemic parameters [299, 300]. The KDIGO 2022 guidelines prioritize SGLT2i as first-line therapy for T2DM with comorbid CKD/obesity-related nephropathy, emphasizing renoprotection over conventional glycemic metrics [301]. The combination use of empagliflozin (EMPA) and topiramate resulted in a significant reduction in body weight and was generally well-tolerated in overweight/obese non-diabetic adults on a calorie-restricted diet [302]. In light of the points put out, more research is necessary to assess the possible benefits of using this combination for long-term maintained weight management.

However, most currently approved anti-obesity drugs are associated with significant adverse effects. Phentermine and amphetamines, for instance, increase cardiovascular risks, including hypertension and arrhythmias [303]. Orlistat, a lipase inhibitor, commonly induces gastrointestinal complications such as steatorrhea and constipation due to impaired fat absorption, with rare cases linked to fatal outcomes [303]. Chronic use of topiramate or phentermine correlates with nephrotoxicity [304, 305], while bupropion and naltrexone have been implicated in renal dysfunction and acute kidney injury, respectively [306, 307]. Orlistat may also provoke tubulointerstitial nephritis [308].

Despite their efficacy, glucagon-like peptide- 1 receptor agonists (GLP- 1 RAs) face limitations due to transient therapeutic effects, high discontinuation rates (driven by nausea and diarrhea), and safety concerns, including pancreatitis, thyroid cancer, gallbladder disorders, and injection-site reactions [309–313]. Furthermore, subcutaneous administration of GLP- 1 RAs necessitates blood–brain barrier (BBB) penetration, which compromises their utility in addressing cognitive aspects of addiction and obesity. Although intranasal delivery has been proposed to enhance brain targeting [314], practical challenges-such as nasal physiological barriers and drug solubility-hinder its clinical translation.

These limitations, coupled with the prohibitive cost and short-term prescribing patterns of newer therapeutics, have intensified interest in alternative strategies, particularly plant-derived compounds and dietary supplements, as adjunctive or primary interventions for obesity management.

#### Nature compounds with potential anti-obesity activity

***Curcumin*** Curcumin, a bioactive polyphenol isolated from the rhizome of Curcuma longa L., demonstrates multifaceted therapeutic properties encompassing anti-inflammatory, anti-proliferative, and redox-modulating activities [152, 315, 316]. Curcumin treatment (1500 mg/day) significantly improved overall β-cell function and reduced both IR and body weight when compared to a placebo (HOMA-β: 136.20 vs 105.19, HOMA-IR: 4.86 vs 6.04, adiponectin: 14.51 vs 10.36, leptin: 9.42 vs 20.66, BMI: 25.94 vs 29.34), with minimal adverse effects, according to a 12-month randomized controlled trial in obese patients with T2DM [317]. For obese patients with T2DM, curcumin therapy may be helpful.

***Genistein*** Legumes like soybeans and soy-rich products contain significant levels of genistein, an isoflavonoid functioning as a selective estrogen receptor modulator (SERM) with pleiotropic metabolic effects [318–320]. Intriguingly, preclinical investigations utilizing gonadectomized murine models subjected to high-fat high-sucrose (HFHS) dietary challenge demonstrated that genistein supplementation effectively ameliorates obesity-associated metabolic perturbations, particularly hepatic steatosis progression and glucose homeostasis dysregulation [321]. While clinical validation in hormone-deficient obese populations remains pending, this phytoestrogen exhibits significant potential as a nutraceutical candidate for mitigating metabolic syndrome components and adiposity-related comorbidities.

***Berberine (BBR)*** *Coptidis Rhizoma*, the rhizome of *Coptis chinensis*, is referred to as Huang Lian in traditional Chinese medicine. It is abundant in bioactive alkaloids, with BBR as its main component. BBR exhibits broad pharmacological effects, including anti-hypertensive, anti-diabetic, anti-adipogenic, anti-inflammatory, antioxidant, and lipid-lowering properties [322, 323]. BBR also exists in other medicinal plants, such as *Berberis aristata*, and *B. vulgaris* [324], and it is metabolized into berberrubine (M1), thalifendine (M2), demethyleneberberine (M3), and jatrorrhizine (M4) [325].

**Fig. 9:**
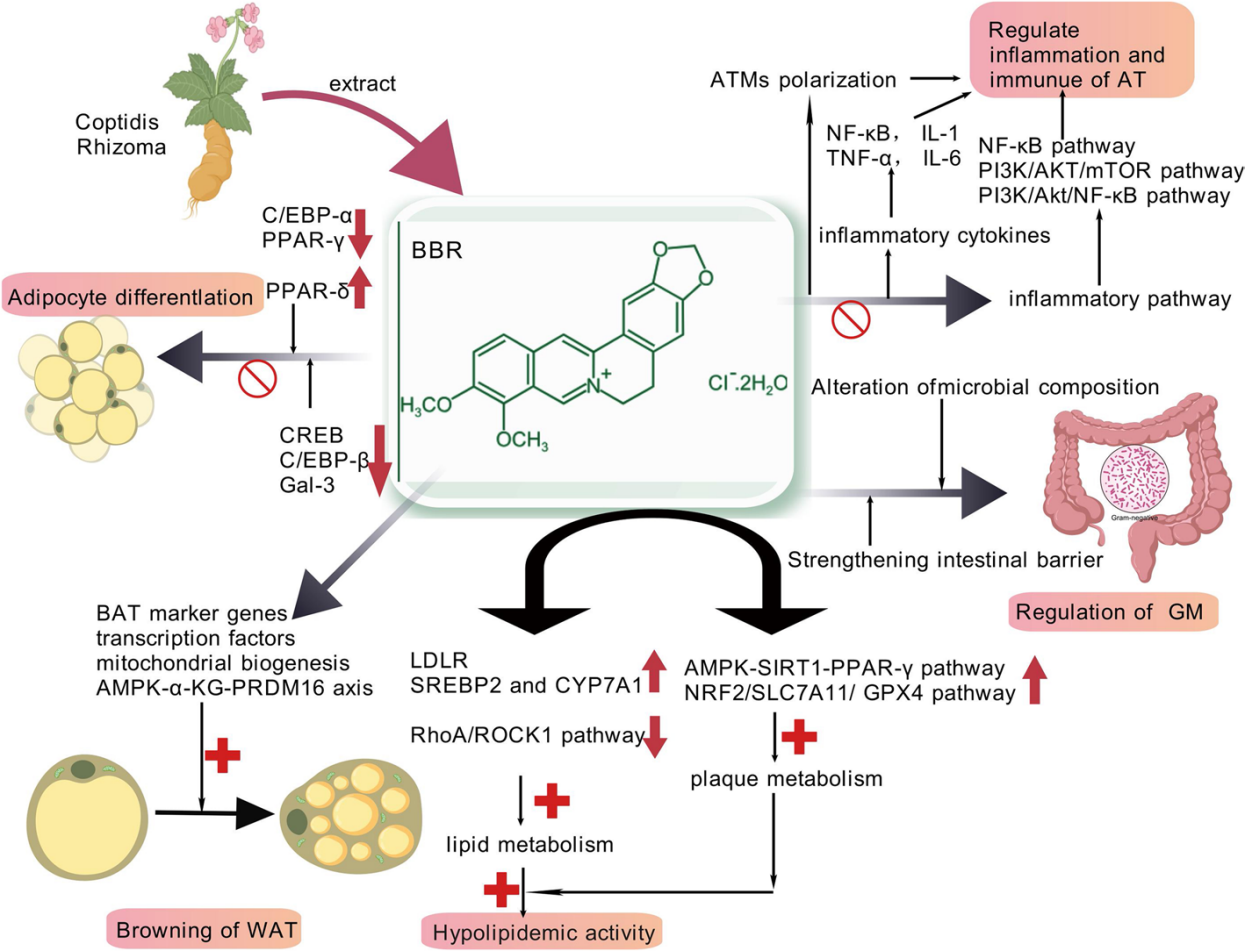
The Mainly Anti-Obesity Mechanisms of BBR. BBR exerts anti-obesity effects through multiple mechanisms: 1) Suppressing adipocyte differentiation by downregulating C/EBP-α, PPAR-γ, and CREB; 2) Promoting browning of WAT via activation of BAT marker genes (e.g., PGC- 1α, UCP1) and mitochondrial biogenesis; 3) Regulating lipid metabolism by upregulating LDLR and Ampk-SIRT1-PPAR-γ pathway; 4) Modulating gut microbiota by increasing Bacteroidetes/Firmicutes ratio and SCFA-producing bacteria; 5) Polarizing adipose tissue macrophages from M1 to M2 phenotype to reduce inflammation; 6) Inhibiting inflammatory pathways (e.g., NF-κB, PI3 K/AKT/mTOR) and cytokines (e.g., TNF-α, IL- 6). Figure 9 was created with BioGDP.com. C/EBP-α, CCAAT/enhancer-binding protein-α; PPAR-γ, peroxisome proliferator-activated receptor γ; CREB, cAMP-response element-binding protein; Gal- 3, Galectin- 3; α-KG, α-ketoglutarate; LDLR, low density lipoprotein receptor; RhoA, Ras homolog gene family member A; NRF2, nuclear factor erythroid 2; SLC7 A11, recombinant solute carrier family 7 member 11; GPX4, glutathione peroxidase 4; GM, gastrointestinal microbiota; ATMs, adipose tissue macrophages. Remarks: All above figures were created with BioGDP.com

Recent researches and preclinical investigations have increasingly emphasized the potential anti-obesity properties of BBR. The mainly potential mechanisms of BBR against obesity are summarized as follows (Fig. 9):

1. Suppression of Adipocyte Differentiation:
  a Inhibits adipogenesis: In 3 T3-L1 adipocytes, BBR down-regulates CCAAT/enhancer-binding protein-α (C/EBP-α) and peroxisome proliferator-activated receptor γ (PPAR-γ), while up-regulating PPAR-δ [320, 325–327].
  b Attenuates cAMP/PKA-mediated signaling: BBR reduces cAMP-response element-binding protein (CREB) and Galectin- 3 signaling, which is a key pathway for its anti-obesity effects [328–334].
2. Adipose Tissue Browning and Metabolic Regulation:
  a Activates BAT thermogenesis: BAT upregulates PGC- 1α, UCP1, PPAR-α, and mitochondrial biogenesis markers (ATPsyn, COXIV, Cyto C) [335–337].
  b Enhances brown adipogenesis: BBR increases PRDM16-driven brown adipogenesis through AMPK-α-ketoglutarate-dependent epigenetic modulation [338].
3. Regulation of lipid metabolism:
  a Regulation of lipid metabolism: BBR regulates the expression of adipokines [339, 340]. It up-regulates LDLR expression by activating AMPK-dependent Raf- 1 and ERK signaling pathway [341–343], up-regulates SREBP2 and CYP7 A1 expression [344–346], and promotes lacteal junction zippering by suppressing the Ras homolog gene family member A (RhoA)/Rho-associated kinase 1 (ROCK1) signaling pathway [347–349].
  b Regulation of plaque metabolism: BBR impedes foam cell formation by activating the AMPK-SIRT1-PPAR-γ pathway which inhibiting the expression of lectin-like oxidized LDL receptor 1 (LOX- 1) [350]. It also stabilizes the plaque by acting as an ACSL4 inhibitor through activitng nuclear factor erythroid 2-related factor 2(NRF2)/recombinant solute carrier family 7 member 11 (SLC7 A11)/glutathione peroxidase 4 (GPX4) pathway [351, 352].
4. Gut Microbiota (GM) Regulation:
  a Alteration of microbial composition: BBR restores the Bacteroidetes/Firmicutes balance, enriches SCFA-producing bacteria, and suppresses bacterial proliferation [351, 353–360].
  b Enhancement of intestinal barrier integrity: BBR modulates the abundance of Akkermansia muciniphila and the IL- 25/mucin- 2 dynamics [361, 362].
5. Anti-Inflammatory Effects in AT:
  a Polarizes AT macrophages (ATMs): BBR polarizes ATMs from the M1 to the M2 phenotype, reducing NF-κB/NLRP3 inflammasome activity [363–370].
  b Suppresses inflammatory pathways: BBR suppresses (NF-κB, PI3 K/AKT/mTOR) and cytokines (TLR4, TNF-α, IL- 6) [371–376]. Similarly, Poulios et al. comprehensively reviewed the mechanisms underlying the anti-obesity effects of key phytochemicals, with BBR being a prominent example [377].

BBR exhibits a favorable safety profile. Although it may cause transient gastrointestinal side effects such as nausea and diarrhea, these often subside with continued use [378]. When co-administered with quercetin, it can alleviate constipation [379]. However, due to its low oral bioavailability [380–382], some innovative strategies are explored:

a Synergistic herbal formulations, such as combined alkaloid extracts, increase bioavailability [383–387].
b Co-crystallization methods, for example, forming BBR-ibuprofen salts, offer a potential solution [360, 388–391].
c Targeted delivery systems such as nanoparticles and liposomes can enhance cellular uptake and bioavailability [153, 278, 392–397].

BBR represents a multifaceted anti-obesity agent with pleiotropic mechanisms, though clinical validation through rigorous RCTs is imperative. Optimizing its pharmacokinetic limitations via advanced formulation technologies could unlock its full therapeutic potential.

### Bariatric surgery

Patients with severe obesity (BMI ≥ 37.5 kg/m^2^) or those who have not responded to medication and lifestyle changes are usually the ones who undergo bariatric surgery [398]. Bariatric surgery, which modifies gut anatomy, significantly impacts food intake and nutrient absorption. This intervention not only facilitates sustained weight reduction but also improves metabolic disorders, obesity-related comorbidities (particularly T2DM), and metabolic syndrome. Additionally, it enhances quality of life and extends survival duration [399–402]. Sleeve gastrectomy (SG), Roux-en-Y gastric bypass (RYGB), adjustable gastric banding (AGB), and biliopancreatic diversion with duodenal switch (BPD-DS) are the four most common bariatric surgeries carried out globally [400, 403]. One anastomosis gastric bypass (OAGB) and single-anastomosis duodenal ileostomy with sleeve gastrectomy (SADI-S) also are two more commonly recommended miainstream techniques [404]. Song et al. demonstrates that SG may ameliorate renal injury and enhance uric acid excretion in HN mice by modulating the AMPK/nuclear factor erythroid 2-related factor 2 (Nrf2) pathway and up-regulating urate transporter ABCG2 transcription [405].

A retrospective analysis involving 498 severely obese patients who underwent SG, RYGB, or OAGB revealed that SG and OAGB were both safe and effective primary surgical options. However, OAGB and RYGB demonstrated superior weight loss outcomes compared to SG [406]. Another study comparing AGB, RYGB, and SG reported total weight loss (TWL) percentages of 36.29%, 31.59%, and 21.07%, respectively, within the first postoperative year [407]. Furthermore, a separate retrospective analysis of over 500 extremely obese patients indicated that BPD/DS yielded the highest TWL (38.4%), followed by RYGB (26.3%) and SG (23.6%). Notably, the 30-day complication rate was significantly higher in the BPD/DS group (12.9%) compared to RYGB (4.7%) and SG (8.7%) [408]. Lucoc’s study included a prospective follow-up of 319 patients who had both LSG and LRYGB (2008–2022) at a tertiary referral center, is consistent with the above conclusions that LRYGB is associated with greater rates of persistent excess weight loss over long-term follow-up [409]. Meanwhile, in a retrospective analysis of patients with a minimum two-year follow-up, Samuel et al. concluded that super-obese patients undergoing LRYGB, as opposed to LAGB and LSG, achieve the best mid-term outcomes in terms of weight loss and resolution of obesity-related comorbidities [410].

However, there are dangers and difficulties associated with bariatric surgery according to growing evidence.Individual-level hurdles to bariatric surgery were found to include fear of surgery, fear of changing own lifestyle, the belief that weight had not reached its ‘tipping point’, worries about dietary modifications, a lack of social support, and fear of influence referral [411]. Despite its potential benefits, the adoption of bariatric surgery has been constrained by several factors, including its invasive nature, substantial financial burden, and the risk of postoperative complications. These limitations have contributed to its relatively restricted application in clinical practice. LSG has a high rate of long-term failure because that one out of three patients will have another bariatric procedure within a decade's time, and half of the patients will gain weight, while up to 90% of patients will occur nutritional deficiencies and a decrease in bone mass over the course of a long-term follow-up [412–415]. Bariatric surgery combined with VD insufficiency is frequent and is anticipated to have a negative effect on the bones [416]. Bariatric surgery also may result in dumping syndrome [417]. Extremely obese people are more likely to experience comorbidities, mortality, surgical problems, and decreased weight loss after bariatric surgery.

For bariatric surgery patients to have successful and long-lasting results, postoperative care techniques are required. The first 24 h of inpatient postoperative treatment are devoted to pulmonary hygiene, early ambulation, intravenous fluid therapy, pain management, supplemental oxygen, and symptomatic management of nausea or vomiting [418]. In the postoperative phase, positive airway pressure treatment can lower the risk of apnea and prevent hypoxic episodes [419–421]. High-quality evidence suggests that the risk of pulmonary embolism and deep vein thrombosis (DVT) can be significantly lowered through a combination of pharmacological interventions and mechanical prophylaxis. Specifically, the administration of unfractionated heparin or low-molecular-weight heparin (LMWH) within 24 h post-surgery, when used alongside intermittent pneumatic compression devices or graduated compression stockings, has been shown to effectively reduce the incidence of these conditions [408, 422–424].

Additionally, bariatric surgery patients should get postoperative nutritional therapy as soon as possible, including enough protein consumption, vitamin and mineral supplements [425–428]. More research is required to determine if moderate doses of VD supplementation (600–3500 IU/day) and high doses (> 3500 IU/day) enhance VD status while having little to no effect on parathyroid hormone levels, according to Chakhtoura et al. [429]. Several studies have shown that Proton pump inhibitors (PPI) significantly lower the incidence of ulcers and gastroesophageal reflux disease [430–432]. Moreover, T2DM patients may additionally require modifications to their anti-diabetic medications due to the possibility of hypoglycemia during surgery period [433, 434]. Endoscopic care of fistulas, leaks, and ulcers has emerged as the first-line treatment when complications arise, and the arsenal of tools and methods is expanding [435, 436]. In addition, White et al. reported that the combination of endoscopic therapy and pharmacologic therapy can address weight recidivism, insufficient weight reduction, or further ameliorate related medical comorbidities [437].

### Emerging therapies

#### Novel medications and targets

***Interleukin- 2 (IL- 2)*** Emerging evidence from murine models of diet-induced adiposity demonstrates the metabolic regulatory potential of low-dose IL- 2 administration. Moon et al. elucidated a dual mechanistic pathway: a. Direct immunomodulatory effects on CD4^+^ T lymphocytes, enhancing regulatory T cell (Treg: CD4^+^, CD25^+^, FoxP3^+^) differentiation while suppressing Th1-mediated gonadal WAT (gWAT) inflammation; b. Neuroimmune crosstalk activation through hypothalamic microglial engagement, stimulating sympathetic outflow that upregulates TGF-β expression concomitant with reductions in pro-inflammatory mediators (IFN-γ, IL- 1β, IL- 6, IL- 8) [438].

***Glucocorticoid (GC)*** GCs are steroid hormones. Both endogenous and exogenous GC excess are detrimental to health as it can result in maladaptive diseases that mimic the metabolic abnormalities brought on by a HFD, such as Cushing’s syndrome [439], hypertension [440], central obesity [441], IR [442], and osteoporosis [32]. In accordance with Zhong’s research, osteoblastic 11β-hydroxysteroid dehydrogenase type 1 (11β-HSD1) is directly linked to obesity, glucose management dysfunction, and bone loss brought on by a HFD. The enzymatic upregulation of 11β-HSD1 demonstrates dual regulatory effects-suppressing osteoblastic glucose utilization and differentiation while amplifying glucocorticoid-mediated repression of Early Growth Response 2 (Egr2) transcription. Pharmacological intervention using DSS, a bone-specific 11β-HSD1 inhibitor, presents novel therapeutic potential for counteracting HFD-associated metabolic dysregulation and osteopenia [443]. Nevertheless, the clinical development of 11β-HSD1 inhibitors is still complicated and unsatisfactory [444, 445]. Finding the precise tissues or cells to target could be an innovative strategy to the development of 11β-HSD1 inhibitors.

***Marine fish oil (FO)*** Marine food that is abundant in long-chain omega- 3 polyunsaturated fatty acids (LC n- 3 PUFA) and long-chain omega- 6 polyunsaturated fatty acids (LC n- 6 PUFA) has been suggested in a number of studies to be a fruitful alternative for lowering obesity and metabolic problems associated with obesity [446, 447]. Furthermore, marine FO offers micronutrients like potassium, iodine, and selenium as well as vitamins A and D [448]. Pradhan et al. have isolated and studied the Tapra FO which was enriched with essential FA, treatment of Tapra FO in the mice displayed anti-obesity impact in terms of decreasing body weight, BMI, serum lipid profiles, leptin and TNF-α in mice model [449]. Marine-derived nutritional interventions, particularly those rich in long-chain omega- 3/omega- 6 LC-PUFAs, exhibit anti-adipogenic properties through leptin signaling interference. Preclinical studies document that Phasa FO supplementation (12.5 mg/kg/day) containing conjugated LC-PUFAs significantly downregulates leptin expression at transcriptional and translational levels, effectively inhibiting adipocyte hyperplasia and lipid accumulation [450]. These findings corroborate marine bioactive compounds as promising candidates for obesity mitigation strategies [451]. Notably, marine natural products (MNPs) demonstrate broad-spectrum biomedical applications, showing therapeutic efficacy against viral pathogens (HIV, SARS-CoV- 2 variants), chronic infections (tuberculosis, H. pylori), and metabolic comorbidities (diabetes, infection-related cardiovascular disorders) [452].

***Penthorum chinense Pursh (PCP)*** PCP, a traditional Chinese medicine, has been used for centuries to relieve the symptoms of excessive alcohol consumption, and treated traumatic damage, edema, and liver disorders such as hepatic viral infections (ALD), NAFLD, and liver fibrosis additionally [453–455]. Hu et al. found that PCP supplementation resulted in reduced body weight and hyperglycemia by decreasing the abundance of Firmicutes and increasing the proportion of Bacteroidetes at the phylum level [456]. Additionally, Hu et al. investigate how PCP treatment improved dyslipidemia and decreased food consumption and obesity. This may be because PCP activates the liver's GLUT2/glucokinase (GCK) expression and lowers hepatic oxidative stress in db/db mice [457]. Besides, there are no specific human experiments to elucidate the anti-obesity mechanism of PCP.

***Myeloid differentiation factor 2 (MD- 2) inhibitors*** Novel MD- 2 inhibitors (MAC28 and 2i- 10) exhibit neuroprotective effects by attenuating MD- 2-toll-like receptor 4 (TLR4)-mediated neuroinflammation. These compounds preserve hippocampal neurogenesis while mitigating obesity-associated cognitive deficits through modulation of microglial activation and oxidative stress markers [458]. Similarly, further clinical studies are supposed to MD- 2 inhibitors as an adjunct to the treatment of obesity.

***Diosgenin (DSG)*** DSG, a naturally occurring steroidal saponin found in a variety of plants, including Solanum and Dioscorea, has a variety of actions in inflammatory illnesses. DSG is also recognized to be beneficial against metabolic problems linked to obesity and IR [459]. Experimental models reveal DSG-mediated suppression of lipogenic regulators (sterol regulatory element-binding protein 1c (SREBP- 1c) and fatty acid synthase (FAS)) with concurrent upregulation of lipolytic enzymes (phospho-AMPK (p-AMPK), phospho-acetyl-coA carboxylase (p-ACC), and carnitine acyl transferase 1 A (CPT- 1 A)), effectively reducing ectopic lipid deposition [460]. In vivo study, the administration of a DSG regimen improved various weight-related outcomes and obesity-related IR by enhancing IRS1/2-PI3 K-Akt signaling pathway activation [461].

***Positive regulatory domain PRDM16*** A β3 adrenergic receptor agonist called mirabegron might boost whole-body energy expenditure and activate human BAT [462, 463]. Nonetheless, Higher dosages may not be clinically used due to possible cardiovascular side effects [464]. PRDM16 is not a direct pharmacological target like GLP- 1 receptor agonist and β3 adrenergic receptor agonist. The mechanism of PRDM16 in AT is as follows: PRDM16 directly activates the thermogenic function of BAT and induces the browning of WAT by binding to the promoter of UCP1 and PPAR-γ coactivator 1α (PGC1α), indirectly regulates AT function by promoting SLIT2 protein secretion and inducing β-hydroxybutyrate (BHB) secretion [465]. According to numerous studies, a number of medications, such as resveratrol [466], rutaecarpine [467], acadesine (AICAR), metformin [468], rosiglitazone [469], and liraglupeptide [470], can reduce obesity and diabetes by altering the expression and function of PRDM16. Nevertheless, PRDM16 is also expressed in cardiac and skeletal muscle. Therefore, there is still more work to be done to target the PRDM16 protein in thermogenic AT in order to battle obesity and the metabolic diseases that are associated with obesity.

***Vanadium compounds*** As long-acting insulin sensitizers, organically derivatized polyoxovanadates (POVs) modified with long-chain aliphatic acids significantly reduce body weight in HFD-fed mice after 8-week administration, notably attenuating adipose tissue accumulation and inflammation. These findings suggest that vanadium-based compounds targeting obesity-associated proteins represent a promising pharmacological strategy for obesity management [471].

***Pancreatic lipase (PL) inhibitors*** Frans et al. demonstrated that tetrahydrocannabinol (THC) and cannabinol (CBN), bioactive extracts from *Cannabis sativa (C. sativa)*, competitively inhibit PL activity. This highlights the potential of C. sativa-derived compounds as novel candidates for developing anti-obesity therapeutics and weight-regulatory agents [472].

Collectively, these advances underscore innovative therapeutic avenues for obesity. However, rigorous preclinical validation and clinical trials remain imperative to evaluate the efficacy, safety, and translational applicability of these approaches in diverse populations.

#### Device-based therapies

The significance of device-based therapies is further highlighted by the complexity and prevalence of obesity and the metabolic problems that accompany obesity [473]. Mobile smart device-based health interventions (mHealth) may offer an appealing and economical strategy for encouraging long-term adaptations of healthier lifestyles, according to a two-arm parallel cluster-RCT [474].

Meanwhile, a medical device based on polyglucosamine polymers (PG) shown a substantial effect on lowering body weight, IR, and cholesterol levels by binding lipids in the upper gastrointestinal tract and decreasing their availability, according to Rondanelli et al.’s innovative and safe treatments for obesity [475]. Additionally, Simvastatin (Sim) encapsulated within PLGA NPs (Sim-NP) was created by Mohaghegh et al. for localized delivery of Sim to ATs for immuno-modulation, which significantly reduced the progression of inflammation linked to obesity, controlled the synthesis of white fat, and improved AT modulation [476].

#### Genetic therapies

Tang et al. demonstrated that adeno-associated virus (AAV)-mediated fat- 1 gene therapy—targeting a fatty acid desaturase that converts omega- 6 to omega- 3 FFAs—ameliorates obesity-induced metabolic dysfunction, cellular senescence, and osteoarthritis by modulating FFA composition [477]. MiRNAs, critical epigenetic regulators, exhibit dynamic expression patterns during adipogenesis: persistently upregulated miRNAs in obesity are suppressed during adipocyte differentiation, whereas downregulated miRNAs in obese individuals are elevated in mature adipocytes, thus highlighting their potential as novel therapeutic targets for obesity [478].

Furthermore, Attia et al. showed that dulaglutide treatment mitigates oxidative DNA damage and hypermethylation in obese animals by restoring the expression of DNA repair genes (e.g., DNMT1, OGG1, and p53), thereby preserving genomic integrityc [479]. Genetic screening for rare obesity-related disorders, informed by clinical insights from pediatric weight management specialists, is essential for optimizing adolescent obesity care [480]. Implementing next-generation sequencing (NGS) to identify variants in Lep, LepR, MC4R, and POMC genes enables timely, genetically guided interventions for non-syndromic early-onset obesity in children and adolescents [481]. Clinical advancements include setmelanotide (IMCIVREETM, Rhythm Pharmaceuticals), an MC4R agonist approved for monogenic obesity disorders (POMC, LepR deficiencies). Ongoing trials explore its efficacy in syndromic obesities (Bardet-Biedl, Alström syndromes) and epigenetic dysregulations of the melanocortin pathway [482].

Likewise, preclinical research suggests that adipose-derived mesenchymal stem cell (ADMSC)-based cell and gene therapy may be a promising treatment option for obesity and its metabolic consequences [483]. The process of transferring a donor's feces to a recipient using a nasogastric tube, colonoscope, enema, capsule, or a combination of these is known as a fecal microbiota transplants (FMTs) [484]. FMTs demonstrates transient metabolic benefits in obesity management, with lean donor FMT inducing short-term (6-week) improvements in microbial butyrogenesis and insulin sensitivity. However, longitudinal analysis (18-week follow-up) reveals microbial community reversion to baseline configurations, underscoring the necessity for sustained intervention protocols [485, 486]. Despite the strength of these findings, additional research with bigger sample sizes and longer duration is needed to ascertain the long-term stability of donor engraftment and related phenotypes.

## Conclusion and prospects

According to the World Obesity Atlas 2024, obesity prevalence among Chinese adults and children continues to rise, positioning obesity as a critical global health challenge across all age groups. The multifactorial pathogenesis of obesity and its complications, coupled with incomplete elucidation of pathophysiological mechanisms by current medical approaches, contributes to suboptimal treatment efficacy and unfavorable prognoses. This review systematically examines obesity pathophysiology through six dimensions: energy balance/metabolic adaptation, hormonal regulation, neural control, inflammation/immune responses, genetic/epigenetic factors, and gut microbiota dynamics. We further analyze mechanisms underlying obesity-related comorbidities and evaluate therapeutic interventions, with particular emphasis on BBR—a natural alkaloid-detailing its pharmacological properties, anti-obesity mechanisms, clinical limitations (notably poor bioavailability and absorption), and recent formulation advancements (e.g., derivatives, eutectic compounds, adipose-targeted delivery systems). This research focuses on the multidimensional pathophysiology exploration (neuroendocrine-immune-metabolic crosstalk), optimization of pharmacological agents (GLP- 1 RAs, dual/triple agonists) and natural compounds (BBR formulation enhancement), and echanistic studies on adipose tissue browning, gut microbiota modulation, and epigenetic regulation.

While preclinical studies demonstrate BBR’s potential in modulating adipose activation and metabolic syndrome, current clinical trials lack obesity-specific endpoints and standardized protocols. In addition, BBR’s low bioavailability and poor oral absorption limit its clinical application. Emerging anti-obesity agents (e.g., GLP- 1 receptor agonists, triple incretin agonists) show superior efficacy, necessitating formulation optimization for BBR to achieve clinical competitiveness. Furthermore, most novel therapeutics remain in preclinical stages, requiring rigorous safety/efficacy validation.

Future obesity-related research should focus more on the following priorities: 1. Translational Development: Clinical validation of preclinical anti-obesity candidates (e.g., IL- 2 analogs, marine-derived compounds) and bioavailability enhancement strategies for phytochemicals (nanodelivery, structural analogs). 2. Precision Medicine: Biomarker discovery for personalized obesity subtyping and treatment, and long-term safety/efficacy studies of novel agents. 3. Preventive Paradigms: Early-life interventions targeting developmental origins of obesity and public health policies addressing obesogenic environments. 4. Therapeutic Innovation: Non-surgical alternatives for high-risk populations (device-based/gene therapies), and combinatorial approaches integrating pharmacotherapy, microbiota modulation, and behavioral interventions.

## Data Availability

Not applicable.
